# An investigation of the association between Juvenile rheumatic disease and growth: a retrospective matched cohort study

**DOI:** 10.1007/s00296-026-06287-x

**Published:** 2026-09-06

**Authors:** Nivethigha Elango, Liza J. McCann, David M. Hughes

**Affiliations:** 1 Population Data Science, Swansea University Medical School, Swansea University, Swansea, UK; 2 Department of Health Data Science, University of Liverpool, Waterhouse Building, Block F, 1-5 Brownlow Street, Merseyside, Liverpool, L69 3GL England; 3 Department of Paediatric Rheumatology, Institute in the Park, Alder Hey Children’s NHS Foundation Trust, Liverpool, UK; 4 Department of Woman and Children’s Health, Institute of Life Course and Medical Sciences, University of Liverpool, Liverpool, UK

**Keywords:** Arthritis, juvenile, Lupus erythematosus, systemic, Dermatomyositis, Growth and development

## Abstract

Childhood-onset rheumatic diseases including Juvenile Idiopathic Arthritis (JIA), Juvenile Systemic Lupus Erythematosus (jSLE), and Juvenile Dermatomyositis (JDM) could impact growth, but this is not well described within the UK population, particularly over the long term. This study aims to compare the growth patterns of patients with JIA, jSLE, and JDM to those of their peers. This population-based cohort study used primary care data from the Clinical Practice Research Datalink (CPRD) with follow up through to March 2023. Patients diagnosed with JIA, JDM and jSLE, were matched with up to five healthy controls by age, sex and general practice. Age and sex standardised Z-scores were calculated for height and weight. Linear mixed models were used to investigate differences in growth patterns over time. Height measurements from 3736, 182 and 111 children with JIA, jSLE and JDM and weight measurements from 5088, 251 and 144 children with JIA, jSLE and JDM respectively were extracted. JIA patients showed significant baseline differences in height compared to healthy controls (Estimate= -0.061, 95% CI 0.002, 0.121) but this initial reduction was countered by an increased rate of catch up growth over time. Similar patterns were observed in jSLE and JDM, although differences were less consistent and largely confined to specific subgroups. Overall, weight trajectories were broadly similar to those of matched controls across all three diseases. Stratified analyses suggested that growth patterns varied according to age at disease onset, with the clearest differences observed in JIA. Children with JIA, jSLE and JDM demonstrated altered longitudinal growth patterns, with the clearest evidence observed in JIA. Growth trajectories varied according to age at disease onset, particularly during the pre and peri-pubertal period, suggesting that the timing of disease onset may influence long-term growth. These findings support the importance of longitudinal growth monitoring, particularly among children diagnosed during key developmental periods.

**Supplementary Information:**

The online version contains supplementary material available at https://doi.org/10.1007/s00296-026-06287-x.

## Introduction

Childhood-onset rheumatic diseases, including Juvenile Idiopathic Arthritis (JIA), Juvenile Systemic Lupus Erythematosus (jSLE) and Juvenile Dermatomyositis (JDM), pose significant health challenges due to their chronic nature and potential for persistent disability. These conditions often manifest at a young age, with the potential to adversely affect growth and development [1–3].

In patients with JIA, jSLE and JDM, growth impairment is a recognised complication, influenced by chronic inflammation, corticosteroid exposure, reduced physical activity, and delayed puberty that disrupts normal growth, leading to stunting, short stature and increased vulnerability to illness [3–8]. Monitoring childhood growth trajectories is therefore essential for assessing disease impact and guiding treatment strategies [9]. “Growth and Development” has been recognised as a key paediatric domain for disease activity and damage assessment in jSLE and JDM [10] yet there is limited evidence available specifically from UK populations.

Although growth patterns in children with JIA, jSLE and JDM have been studied, existing research is often limited by small sample sizes and short follow-up periods. Consequently, findings across studies are often inconsistent, with many lacking sufficient power to detect meaningful differences in growth outcomes [11]. Moreover, much of the literature has focused on affected populations within hospital cohorts (which may reflect more severe or longstanding disease) with limited comparison to the general population, constraining interpretation of the broader impact of these conditions on growth trajectories.

This study aims to address these gaps through a retrospective matched cohort study examining growth patterns in children with JIA, jSLE, and JDM. We aim to use electronic health records to identify a large cohort of patients with juvenile rheumatic disease. By analysing height and weight over time and comparing these patterns to general population, we aim to describe the impact that juvenile rheumatic disease has on both height and weight. Understanding these growth patterns is important for evaluating disease burden and informing clinical management.

## Methods

### Data source

This study used data from the Clinical Practice Research Datalink (CPRD), a large UK primary care database containing anonymised electronic health records on over 60 million patients (collected over the history of CPRD), with more than 18 million currently registered patients [12]. Patients with implausible diagnosis dates recorded prior to their date of birth were excluded.

### Study design

This was a retrospective matched cohort study, reported in accordance with the Strengthening the Reporting of Observational Studies in Epidemiology (STROBE) guidelines.

### Study population

Patients with clinical codes for JIA, jSLE, and JDM were identified using the SNOMED codes provided in Supplementary Tables 1–3. Our clinical code lists were derived from previously reported code lists for CPRD [13, 14] and the HDRUK Phenotype library (https://phenotypes.healthdatagateway.org). Patient registration records were available from February 1969, and data were extracted in March 2023. An open cohort design was used, with follow-up until deregistration, death, or the study end date (March 2023). Eligible patients were required to have at least one year of CPRD registration prior to diagnosis and to be ≤ 18 years of age at onset. Each case was matched exactly to up to five controls without rheumatic diseases by age (in years), sex, and general practice. It was not possible for each case to be matched to five controls, so some cases had fewer matched controls, explaining the slight differences between groups observed in Tables 1 and 2. For controls, the index date corresponded to the diagnosis date of their matched case.

### Outcomes

The primary outcomes were height (cm) and weight (kg). We converted these absolute values to age and sex standardised z-scores using the UK 1990 growth reference (UK90) LMS method. This allowed conversion of all heights and weights measured up to the age of 20. We did not consider any observations after this point for each individual. Repeated measurements of these outcomes were used to construct growth trajectories following diagnosis. SNOMED codes used to assess height and weight measurements are reported in Supplementary Tables 4 and 5. Data were not available for exact diagnosis date and we used the first recorded clinical code per patient as a proxy of diagnosis date. Height or weight observations dated before birth or prior to the first diagnosis were removed, with first diagnosis code serving as the baseline. Only patients with at least two valid height or weight measurements were retained, as a minimum of two observations is required to meaningfully assess growth changes. Because the study focused on childhood-onset rheumatic diseases, individuals diagnosed after the age of 18 years (16 years for JIA) were excluded. Observations where the age and sex standardised z-scores fell below − 3 or above 4 were removed as implausible values.

### Covariates

Key exposure variables included age at diagnosis, defined as the interval between date of birth and first recorded diagnosis, and pubertal stage at diagnosis, categorised as pre-pubertal (≤ 7 years), peri-pubertal (8–13 years), or adolescent (14–18 years) [15]. Time since diagnosis was defined as the interval between diagnosis and each growth measurement, and time to first post-diagnosis measurement as the interval between diagnosis and the first available growth observation. Additional covariates included sex, ethnicity and diagnosis group (case versus control), and were selected based on previous literature identifying them as significant predictors of growth outcomes [16–18].

### Statistical analysis

Linear mixed models (LMMs) were used to assess growth trajectories, implemented in R using the ‘*lme4’* package [19]. Models included random intercepts, and a random slope to account for correlation between repeated measurements of height or weight on the same individual and to capture individual variation in baseline height or weight and in growth rates. We also fit linear mixed models to the standardised z-scores for height and weight using the same approach. The random effects in these models were assumed to jointly follow a multivariate normal distribution with mean 0, and an unstructured covariance matrix. Models were adjusted for sex, pubertal stage at diagnosis and ethnicity. Interactions between diagnosis group and time since diagnosis were examined to evaluate differential growth trajectories.

To address potential non-linearity in height and weight trajectories, polynomial and spline terms were tested, and model fit was compared using the Akaike Information Criterion (AIC) and Bayesian Information Criterion (BIC). Based on this we used B-splines in all our reported models to capture non-linear change over time in height and weight. We used cubic splines with interior knots selected automatically at appropriate quantiles of the time since diagnosis and boundary knots placed at the minimum and maximum observed values. Splines were assessed visually and model residuals and estimated random effects were assessed with Q-Q plots to determine normality. Results are reported as estimates with 95% confidence intervals, and statistical significance was defined as *p* < 0.05.

Subgroup analyses were conducted to stratify growth trajectories by biological sex and pubertal stage. Missing data were evaluated prior to modelling. There were no missing values for key variables such as age at diagnosis and time since diagnosis. Although repeated measurements can be affected by missingness, LMMs are robust to missing observations in longitudinal data [20], therefore, no imputation was undertaken. Data handling and analysis were conducted using R version 4.2.1.

The main results and discussion are based on analysis of age- and sex-standardized height and weight z-scores, the preferred measures for assessing paediatric growth. Corresponding analyses using absolute height and weight measurements are provided in the Supplementary Material (Supplementary Tables 6–17). Although some differences were observed between analysis based on absolute measurements and z-scores, the latter are presented in the main manuscript because they account for expected growth according to age and sex.

### Ethical considerations

The study protocol and access to the CPRD data were approved by the Independent Scientific Advisory Committee (Study protocol 23_002606). The study description can be found at https://www.cprd.com/approved-studies/examining-comorbidities-patients-childhood-onset-rheumatic-diseases-juvenile. This study is based in part on data from the Clinical Practice Research Datalink obtained under licence from the UK Medicines and Healthcare Products Regulatory Agency. The data is provided by patients and collected by the NHS as part of their care and support. The interpretation and conclusions contained in this study are those of the authors alone.

## Results

The height cohort had 3736 children with JIA, 182 with jSLE and 111 with JDM with at least two height measurements. The weight cohort included 5088 children with JIA, 251 with jSLE, and 144 with JDM who had at least two weight measurements before the age of 21. Across all cohorts, females were more common than males and the majority of participants were of white ethnicity. The mean age at diagnosis varied by condition, with JIA and jSLE cases typically diagnosed in later childhood or early adolescence (10.6 and 11.8 years respectively, Table 1), while JDM cases were younger at diagnosis (8.5 years). Baseline height and weight were significantly lower in cases compared with matched controls across all cohorts. However, weight Z-scores did not show evidence of statistically significant differences. Full patient characteristics are presented in Table 1 for height cohort and Table 2 for weight cohort. The distribution of timings of measurements of height and weight is shown in Supplementary Fig. 1.

**Table 1:** Baseline Characteristics of the Height cohort

Variable		Juvenile Idiopathic Arthritis			Juvenile Systemic Lupus Erythematosus			Juvenile Dermatomyositis		
		Control	Cases	*p*-value	Control	Cases	*p*-value	Control	Cases	*p*-value
N (%)		6337 (62.9)	3736 (37.1)		252 (58.1)	182 (41.9)		135 (54.9)	111 (45.1)	
Age at diagnosis (years), Mean (sd)		10.5 (5.0)	10.6 (4.9)	0.402	11.9 (5.0)	11.8 (4.4)	0.843	7.8 (4.1)	8.5 (4.3)	0.21
Pubertal Stage N(%)	Adolescent	1911 (30.2)	1128 (30.2)	0.374	106 (42.1)	70 (38.5)	0.272	10 (7.4)	12 (10.8)	0.3
	Peri-pubertal	2393 (37.8)	1455 (38.9)		92 (36.5)	80 (44.0)		49 (36.3)	47 (42.3)	
	Pre-pubertal	2033 (32.1)	1153 (30.9)		54 (21.4)	32 (17.6)		76 (56.3)	52 (46.8)	
Ethnicity	White	5326 (84.0)	3185 (85.3)	0.126	201 (79.8)	111 (61.0)	< 0.001	106 (78.5)	88 (79.3)	0.371
	Asian	353 (5.6)	193 (5.2)		23 (9.1)	34 (18.7)		16 (11.9)	9 (8.1)	
	Black	147 (2.3)	63 (1.7)		9 (3.6)	14 (7.7)		7 (5.2)	4 (3.6)	
	Other	511 (8.1)	295 (7.9)		19 (7.5)	23 (12.6)		6 (4.4)	10 (9.0)	
Sex	Male	1800 (28.4)	1137 (30.4)	0.032	51 (20.2)	36 (19.8)	1	35 (25.9)	36 (32.4)	0.327
	Female	4537 (71.6)	2599 (69.6)		201 (79.8)	146 (80.2)		100 (74.1)	75 (67.6)	
Time since diagnosis at first height measurement (years) Median (IQR)		3.5 (3.5)	2.0 (2.7)	< 0.001	3.0 (3.2)	1.8 (2.6)	< 0.001	4.7 (4.1)	2.2 (3.2)	< 0.001
Height (cm) Mean (sd)		155.0 (20.1)	149.1 (23.7)	< 0.001	157.1 (19.1)	152.1 (19.6)	0.008	149.5 (21.2)	140.4 (24.4)	0.002
Height Z score Mean (sd)		0.2 (1.1)	0.1 (1.2)	< 0.001	0.2 (1.1)	-0.1 (1.2)	0.001	0.3 (1.1)	0.1 (1.2)	0.3
Number of observations Mean (sd)		3.3 (2.3)	4.4 (3.9)	< 0.001	3.2 (2.1)	5.4 (4.7)	< 0.001	3.7 (2.3)	5.6 (4.5)	< 0.001

**Table 2:** Baseline characteristics of the Weight cohort

Variable		Juvenile Idiopathic Arthritis			Juvenile Systemic Lupus Erythematosus			Juvenile Dermatomyositis		
		Control	Cases	*p*-value	Control	Cases	*p*-value	Control	Cases	*p*-value
N (%)		8541 (62.7)	5088 (37.3)		368 (59.5)	251 (40.5)		180 (55.6)	144 (44.4)	
Age at diagnosis (years) Mean (sd)		11.0 (5.1)	11.2 (5.0)	0.009	12.4 (5.0)	12.6 (4.3)	0.479	8.2 (4.3)	8.8 (4.4)	0.206
Pubertal Stage N(%)	Adolescent	2996 (35.1)	1829 (35.9)	0.027	173 (47.0)	116 (46.2)	0.096	19 (10.6)	20 (13.9)	0.54
	Peri-pubertal	3015 (35.3)	1861 (36.6)		124 (33.7)	101 (40.2)		68 (37.8)	57 (39.6)	
	Pre-pubertal	2530 (29.6)	1398 (27.5)		71 (19.3)	34 (13.5)		93 (51.7)	67 (46.5)	
Ethnicity	White	7199 (84.3)	4331 (85.1)	0.204	299 (81.2)	156 (62.2)	< 0.001	139 (77.2)	117 (81.2)	0.43
	Asian	468 (5.5)	269 (5.3)		31 (8.4)	41 (16.3)		23 (12.8)	12 (8.3)	
	Black	197 (2.3)	91 (1.8)		8 (2.2)	19 (7.6)		8 (4.4)	4 (2.8)	
	Other	677 (7.9)	397 (7.8)		30 (8.2)	35 (13.9)		10 (5.6)	11 (7.6)	
Sex	M	2015 (23.6)	1352 (26.6)	< 0.001	60 (16.3)	41 (16.3)	1.0	45 (25.0)	46 (31.9)	0.209
	F	6526 (76.4)	3736 (73.4)		308 (83.7)	210 (83.7)		135 (75.0)	98 (68.1)	
Time since diagnosis at first weight measurement (years) Median (IQR)		3.4 (3.5)	2.0 (2.7)	< 0.001	2.8 (3.2)	1.6 (2.3)	< 0.001	4.8 (4.1)	2.3 (3.3)	< 0.001
Weight (kg) Mean (sd)		53.6 (19.4)	50.1 (21.2)	< 0.001	56.3 (19.3)	53.1 (18.9)	0.041	48.7 (20.2)	41.2 (19.0)	0.001
Weight Z-score		0.4 (1.3)	0.4 (1.3)	0.728	0.5 (1.4)	0.4 (1.4)	0.384	0.4 (1.3)	0.4 (1.4)	0.561
Number of observations Mean (sd)		3.8 (2.8)	4.9 (4.7)	< 0.001	3.6 (2.4)	6.1 (6.0)	< 0.001	3.9 (2.6)	5.9 (5.3)	< 0.001

### Juvenile idiopathic arthritis

Children with JIA exhibited significant growth deficits compared with controls. Adjusted linear mixed models demonstrated that, on average, individuals with JIA were shorter than their healthy peers (reduction of 0.061SD, 95% CI 0.002,0.121, Supplementary Table 18) at baseline. No statistically significant baseline differences were observed for weight between the groups (Supplementary Table 20).

**Fig. 1:**
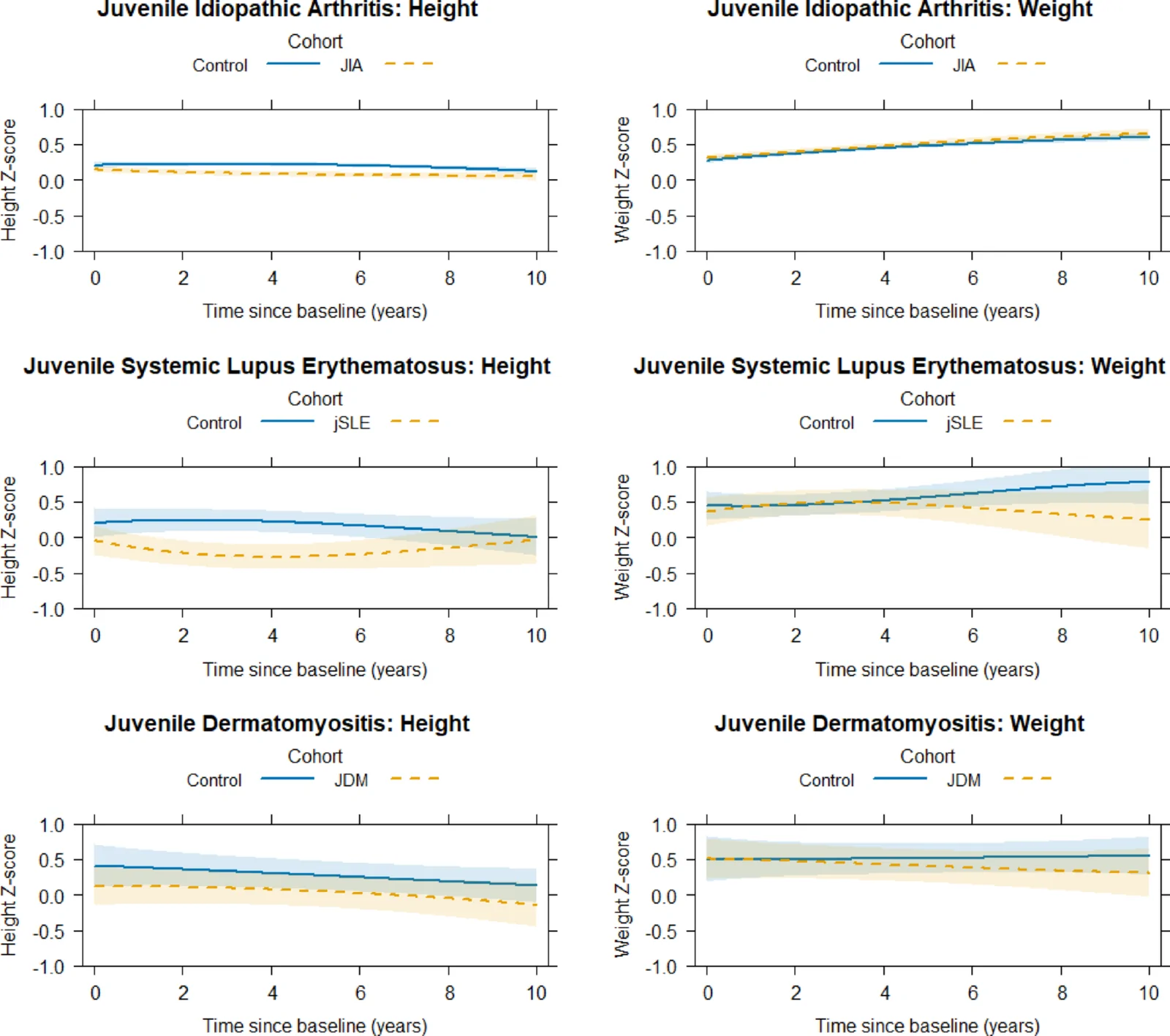
Impact of juvenile disease on model predicted height and weight Z-score trajectories for juvenile rheumatic disease groups and matched controls

Some significant interaction between time since diagnosis and JIA was observed for height z-scores, suggesting that patients with JIA increased in height over time faster than healthy controls. This shows that whilst children with JIA are generally shorter than matched controls at the point of diagnosis, the difference in height between the groups decreased over time. Further, with regards to weight, a similar pattern of change over time was observed, with weight trajectories in individuals with JIA becoming more similar to those of matched controls over follow-up (Fig. 1).

#### Sex-specific differences

**Fig. 2:**
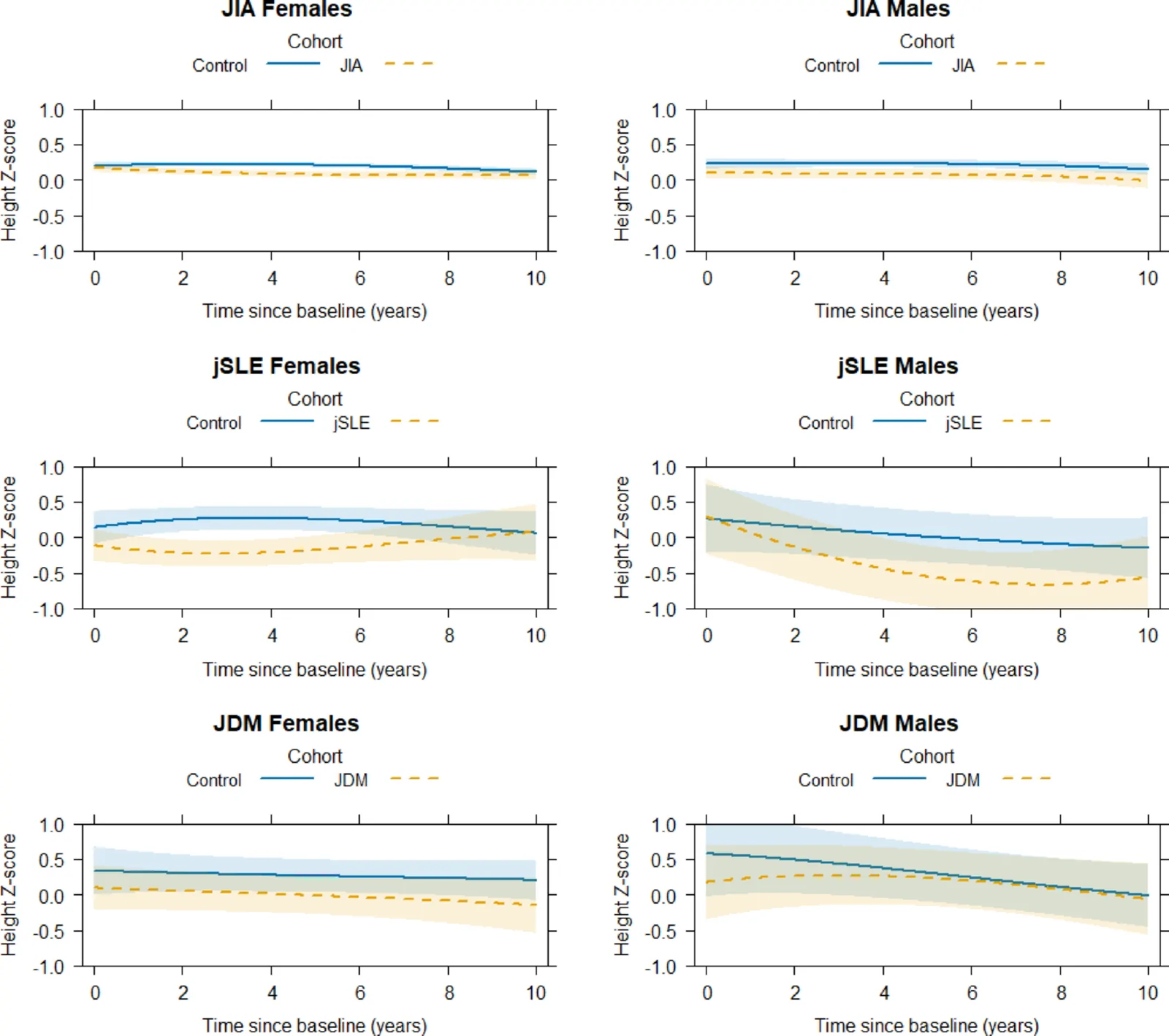
The impact of sex on model predicted height Z-score trajectories for juvenile rheumatic disease groups and matched controls

Females with showed no evidence of reduction in height relative to their age, but a statistically significant reduction was observed for males (Supplementary Tables 18 and Fig. 2). No differences in weight were observed between males or females with JIA compared to healthy controls (Supplementary Tables 20 and Supplementary Fig. 11).

#### Pubertal stage–specific differences

**Fig. 3:**
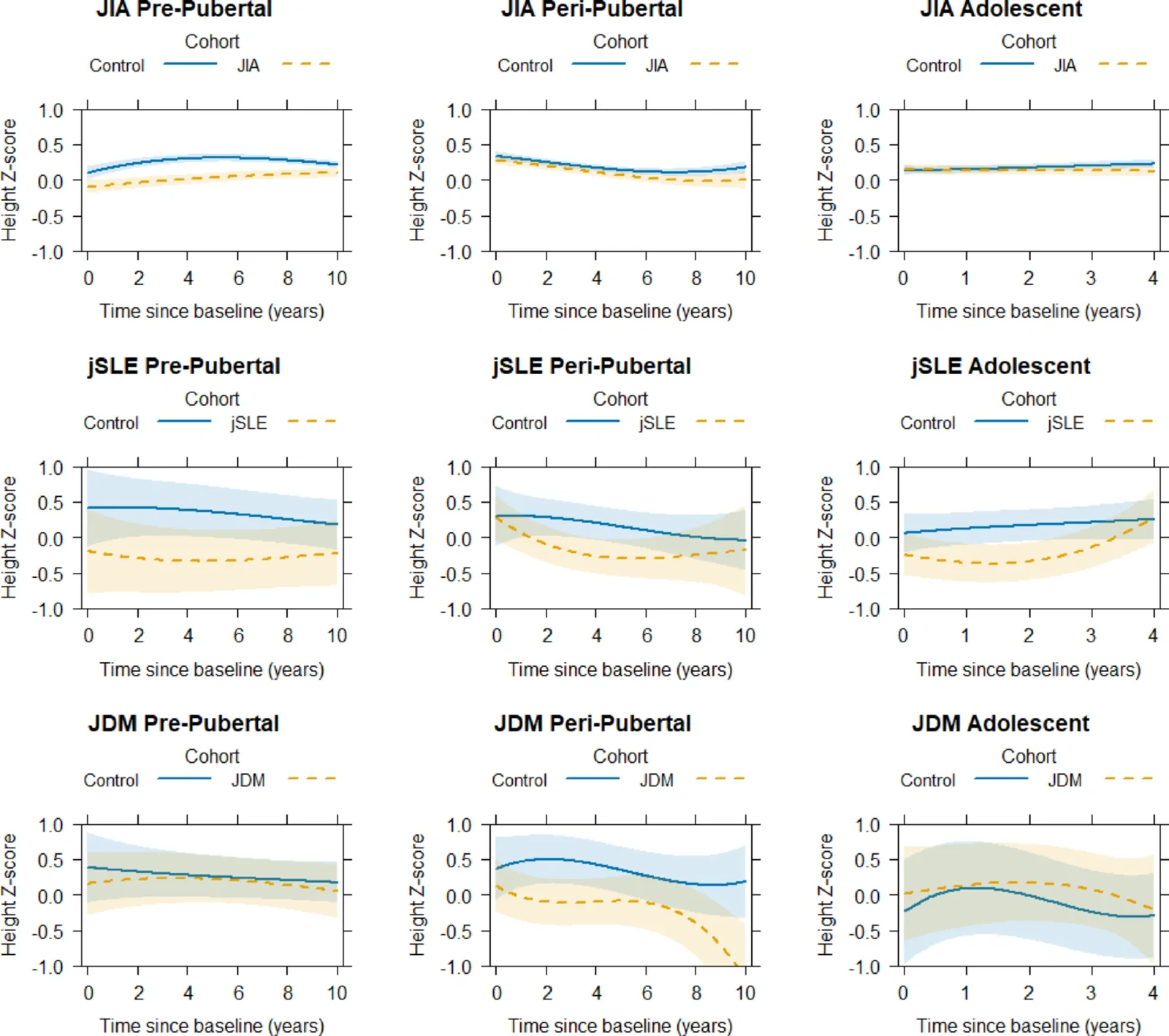
The impact of pubertal stage at diagnosis of juvenile disease on model predicted height Z-score trajectories for juvenile rheumatic disease groups and matched controls

When stratified by pubertal stage, small but significant reductions in height were evident only among children diagnosed before puberty (Estimate = -0.197, 95% CI 0.07, 0.33, Supplementary Tables 19 and Fig. 3).

Similarly, statistically significant reductions in weight were observed for children with JIA compared to healthy controls for those who were diagnosed during pre-pubertal stage (Estimate = -0.20SD, 95%CI 0.08, 0.322, Supplementary Tables 21 and Supplementary Fig. 13). Conversely, children with JIA diagnosed during adolescence had slightly higher weight than controls (0.11SD increase, 95%CI 0.02, 0.20).

### Juvenile systemic lupus erythematosus

No statistically significant baseline differences in height or weight were observed for children with jSLE (Fig. 1, Supplementary Tables 22 and 24 respectively).

#### Sex-specific differences

No significant sex-specific differences in height or weight were observed (Supplementary Tables 22, and 24, Fig. 2 and Supplementary Fig. 11).

#### Pubertal stage–specific differences

No height differences were observed according to pubertal stage at diagnosis (Supplementary Table 23). Children with jSLE diagnosed before puberty were 0.92SDs lighter than matched controls in Z score models (95%CI 0.16, 1.67, Supplementary Table 25), but no other weight differences were observed in the analysis stratified by pubertal stage (Supplementary Table 25).

### Juvenile dermatomyositis

No statistically significant differences in height were observed when comparing children with JDM to controls or in subgroup analysis based on sex (Supplementary Table 26). In the analysis according to pubertal stage, although no baseline difference in height z-scores was observed among children diagnosed during the peri-pubertal stage, growth trajectories differed over follow-up, with children with JDM exhibiting progressively lower height trajectories than matched controls (Fig. 3, Supplementary Table 26).

Children with JDM diagnosed during adolescence were lighter at baseline than controls (1.36SD reduction, 95%CI 0.27,2.42, Supplementary Table 29). Statistically significant interaction terms between JDM and time since diagnosis indicates that children with JDM diagnosed during adolescence increase in weight faster than controls (Supplementary Fig. 13, Supplementary Table 29). However, these results should be interpreted with caution due to the low numbers of children diagnosed with JDM during adolescence (Table 2).

### Catch-up growth

Linear mixed models showed baseline differences between children with JIA and controls that decreased over time. We further investigated final height and weight in a subset of our cohort who had a height or weight measurement between the ages of 18 and 20.

Children with JIA had lower height z-scores beyond age 18, though no statistically significant difference was observed for either comparison in the jSLE and JDM cohorts (Supplementary Table 30, Fig. 1). Also, children with JIA tended to be heavier beyond the age of 18 than controls (Supplementary Table 31). No statistically significant differences were observed for jSLE or JDM (Fig. 1 and Supplementary Fig. 8).

Taken together this suggest that whilst some children with juvenile rheumatic disease may initially have slower growth than controls, but these differences generally diminish over time, and in some cases, by the time adulthood is reached, children with juvenile rheumatic disease have overtaken controls (particularly in weight).

## **Discussion**

This study contributes valuable population-based evidence to the existing literature on the impact on growth of chronic childhood rheumatic diseases. As one of the largest retrospective longitudinal studies conducted in the UK, it offers insights into the long-term growth trajectories of the affected children, aiding in management for healthcare professionals and patients/families.

Children with JIA demonstrated the clearest evidence of impaired linear growth, being shorter at the point of diagnosis, followed by evidence of catch-up growth over time. Similar pattern were observed for children with jSLE and JDM although these results were not statistically significant, possibly due to lower sample size, and hence reduced power. This pattern is consistent with previous literature in JIA, jSLE and JDM, where inflammatory burden, corticosteroid exposure and disease activity have been associated with impaired linear growth, but partial recovery in growth trajectories has also been observed in some cases following improved disease control [2, 6, 17, 18]. However, our findings extend previous work by demonstrating that, although catch-up growth occurs across conditions, the extent and pattern of growth impairment differed between diseases when compared with matched individuals from the general population over long-term follow-up.

Weight differences were less apparent across all three conditions. In our cohort, children with JIA showed little difference in weight overall. However, in stratified analyses, children with JIA during adolescence (> 13 years) had slightly higher weight relative to peers and demonstrated consistently higher longitudinal weight trajectories over follow-up. This finding is similar to previous works that describes a disrupted body composition profile in JIA, characterised by reduced lean mass and relative adiposity, reflected in higher BMI and increased obesity prevalence [2, 11, 21]. This may indicate the importance of closer monitoring of weight in individuals diagnosed later childhood and adolescence, particularly as this age range coincides with pubertal growth, although further investigation is required to determine the underlying mechanism.

Previous studies reported increases in BMI during follow-up, typically peaking around 6 months after diagnosis and remaining above baseline at the final study visit (26 months) [5, 17, 18]. Our study observed no significant difference in weight at diagnosis in both jSLE and JDM cohorts and they both had largely similar long-term weight trajectories compared with their peers. However, children with JIA and jSLE who were diagnosed before puberty (< 8 years) had lower weight at baseline, although these differences diminished over time. Similarly, adolescents with JDM were lighter at baseline but demonstrated a more rapid increase in weight during follow-up, with trajectories converging towards those of matched controls. These differences may be due to methodological differences between studies. Previous studies were generally based on smaller disease cohorts with relatively short follow-up, potentially capturing the immediate/early impact of disease and describing changes within affected individuals, whereas our study compared growth trajectories with those of the general population over a longer follow-up period.

A higher proportion of females than males has been consistently reported across childhood rheumatic diseases in previous studies and this was also seen in our study (Table 1) [1, 4, 5, 17, 21–23]. We found that males with JIA had significantly lower baseline height while such difference was not observed among females. However, these sex-specific findings were not consistent across JIA, jSLE and JDM, suggesting that growth variation may be more strongly influenced by disease-specific factors and developmental timing than by biological sex alone. This could also be due to smaller number of males in each subgroup, reflecting the recognised female predominance in juvenile rheumatic diseases.

Across JIA, jSLE and JDM, evidence relating to the effect of pubertal stage at disease onset to linear growth impairment remains heterogeneous. In this study, reduced height and weight was primarily observed among children with JIA diagnosed before puberty (< 8 years). Several studies in juvenile rheumatic disease have associated pre-pubertal onset with poor height outcomes [2, 5, 16, 18]. Rygg et al. [18] demonstrated that females with jSLE with disease onset before 8 years had persistently reduced height trajectories over follow-up, while Balci et al. [5] similarly reported greater growth failure among females with disease onset around 8–12 years of age. Evidence across all three conditions also suggests that the peri-pubertal period may represent a particularly vulnerable developmental window for growth disruption. Polito et al. [24] reported greater loss of height during puberty than before puberty in JIA, while Heshin-Bekenstein et al. [25] observed that onset of jSLE during peak pubertal growth periods had a marked impact on final adult height. Rygg et al. further demonstrated that the greatest decline in height trajectories occurred among peri-pubertal females aged 8–13 years, despite more severe baseline deficits in pre-pubertal groups [18]. Similarly, our findings for peri-pubertal-onset JDM are consistent with those of Nordal et al., who reported that growth impairment was most evident among individuals in intermediate pubertal Tanner stages, whereas pre-pubertal females appeared less affected [17].

### Relevance of the findings

These findings have important clinical implications:

Growth trajectories varied according to disease type and developmental stage at diagnosis, with some differences becoming less pronounced over time while others emerged only during longer-term follow-up. For example, children diagnosed with JDM during peri-pubertal stage (around 8–13 years) showed no difference in height at diagnosis compared with controls, but developed progressively lower height trajectories during follow-up. These findings highlight that assessment at diagnosis or during the early stages of disease may not fully capture the long-term impact on growth and highlight the importance of continued longitudinal growth monitoring.

Although growth impairment was not universal across all paediatric rheumatic diseases, children diagnosed during particular age groups appeared more susceptible to altered patterns of growth. For example, pre-pubertal-onset JIA was associated with the greatest impairment in linear growth, with similar baseline deficits in weight observed among children with pre-pubertal-onset JIA and jSLE. Together, these findings suggest that growth should be interpreted within the context of both age at disease onset and pubertal development, supporting a more individualised approach to growth surveillance. As pubertal stage in this study was approximated using age at disease onset, future longitudinal studies incorporating direct measures of pubertal development are needed to better understand how pubertal maturation impacts long-term growth outcomes.

Although several growth trajectories showed evidence of convergence towards those of the general population over time, these findings represent average population-level patterns rather than individual outcomes. Some children may continue to experience persistent growth impairment despite the overall improvements observed across the cohort. Consequently, continued growth surveillance remains important, particularly for children diagnosed during developmental periods that appear more vulnerable to altered growth trajectories.

Finally, while anthropometric measures such as weight and BMI remain useful indicators of growth, they may not fully reflect changes in body composition. In our study, adolescents with JIA demonstrated persistently higher weight trajectories over time and at diagnosis. Future studies incorporating assessment of body composition, including lean and fat mass (particularly in children with adolescent-onset JIA), alongside disease activity and treatment exposure, may provide a more complete understanding of growth, physical development, and the longer-term metabolic consequences of paediatric rheumatic diseases.

### Limitations

Despite its notable strengths, this study has some limitations. The absence of data on disease severity scores and treatment regimens with substantial changes in treatment strategies over time, such as early use of DMARDs or biologics limits our ability to fully elucidate the relationship between disease activity, treatment exposure, and growth outcomes. We also did not have data on a child’s nutrition or physical activity levels. For these reasons we cannot directly attribute differences in height and weight to juvenile rheumatic disease itself. We only show that there are differences in children who have juvenile rheumatic disease, and leave for future research assessment as to which, if any of the above factors may most strongly explain or drive these differences.

While our study is one of the largest longitudinal comparison of patients with jSLE or JDM to matched controls, the overall numbers are still not large, which limits the power to detect significant reductions in height or weight, particularly in subgroup analyses. Subgroup analyses in particular should be interpreted with caution, and with awareness of multiple testing issues.

We do not have access to the actual date of diagnosis of juvenile rheumatic disease. As a result, some patients may have had longer time periods between the start of their disease and our baseline, which was taken to be the first recorded code for juvenile rheumatic disease. This may have influenced height and weight measurements. However, it is likely that for most people their first recorded code in a GP record was reasonably close to the actual diagnosis of their disease. Similarly we do not have exact data on pubertal stage at diagnosis and are basing this on the child’s age at first recorded juvenile rheumatic code so our results about pubertal stage should be interpreted with caution.

The retrospective nature of the study, although often pragmatically necessary for studying rare conditions like JDM and jSLE, presents challenges. There are potential inaccuracies in the coding of patient data in the Clinical Practice Research Datalink (CPRD) by General Practitioners, and discrepancies between the recorded date of diagnosis and the actual disease onset date. We have tried to limit the risk of misclassification of juvenile rheumatic disease, by using validated codelists developed and used in prior research by other clinical teams. Patients may remain asymptomatic or not present to healthcare services for extended periods, leading to delays in diagnosis and monitoring. The reliance on primary care data alone poses another limitation, as some conditions may be underreported or missed if they are primarily diagnosed and managed in secondary care settings. By requiring two observations of height or weight for inclusion in each cohort, we may introduce a selection bias that favours children with greater health seeking utilisation. This is an unavoidable restriction in order to perform a longitudinal analysis but should be considered when interpreting our findings. Selection bias towards generally more ill patients is a known limitation of research in electronic health records such as CPRD.

The retrospective nature of our study, considering largely opportunistic data collection prevents us from being able to assess the influence of treatment and disease severity on growth in children with rheumatic disease. Whilst a prospective study with carefully chosen disease groups and controls would be ideal, our study makes a contribution to the evidence of growth differences in juvenile rheumatic disease, that provides an evidence base to be developed in future, more ideal studies.

In conclusion, our study extends the existing evidence base by providing a large UK population-based comparison of longitudinal growth in juvenile rheumatic diseases. Although the magnitude of growth impairment differed across JIA, jSLE and JDM, growth trajectories varied according to age at disease onset and may reflect important periods of pubertal development. These findings emphasise the need for longitudinal growth surveillance alongside optimised disease control to minimise long-term impacts on physical development.

Future studies should incorporate large-scale prospective follow-up with detailed characterisation of treatment regimens, nutritional status, and lifestyle factors across disease groups. Integrating a life-course approach to capture growth trajectories alongside the development of comorbidities will be critical to improving understanding of the cumulative and long-term burden of paediatric rheumatic disease.

## Supplementary Information

Below is the link to the electronic supplementary material.

Supplementary Material 1

## Data Availability

We are not permitted to directly share the data used in this study. The data are available directly from CPRD. The code lists we have used are available in the Supplementary material.

## Declarations

### Disclaimer

This manuscript represents original work that has not been published previously and is not under consideration for publication elsewhere. No artificial intelligence (AI) tools were used in the design, analysis, interpretation of the data, or preparation of this manuscript. All scientific content, analyses, interpretations, and conclusions were developed and verified by the authors, who take full responsibility for the integrity and accuracy of the work.
