# Supplementary material for: An investigation of the association between Juvenile rheumatic disease and growth: a retrospective matched cohort study
Source: Rheumatol Int. 2026 Sep 6;46(9):257. doi: 10.1007/s00296-026-06287-x (PMC13547155; doi:10.1007/s00296-026-06287-x)
Supplement: Supplementary file 1 [file 296_2026_6287_MOESM1_ESM.docx]

This Supplementary Material includes additional figures and tables supporting the analysis presented in the main manuscript, together with the SNOMED code lists used to define juvenile idiopathic arthritis (JIA), juvenile-onset systemic lupus erythematosus (jSLE), juvenile dermatomyositis (JDM), and the identification of height and weight measurements.

**FIGURES:**

Figure 1: Distribution of timing of height and weight measurements

Figure 2: Height trajectories for JIA cases and matched controls. The red line shows a LOESS smoother of the average trajectory.

Figure 3: Weight trajectories for JIA cases and matched controls. The red line shows a LOESS smoother of the average trajectory.

Figure 4: Height trajectories for JDM cases and matched controls. The red line shows a LOESS smoother of the average trajectory.

Figure 5: Weight trajectories for JDM cases and matched controls. The red line shows a LOESS smoother of the average trajectory.

Figure 6: Height trajectories for jSLE cases and matched controls. The red line shows a LOESS smoother of the average trajectory.

Figure 7: Weight trajectories for jSLE cases and matched controls. The red line shows a LOESS smoother of the average trajectory.

Figure 8: Impact of juvenile disease on model predicted height and weight trajectories for juvenile rheumatic disease groups and matched controls

Figure 9: The impact of sex on model predicted height trajectories for juvenile rheumatic disease groups and matched controls.

Figure 10: The impact of sex on model predicted weight trajectories for juvenile rheumatic disease groups and matched controls.

Figure 11: The impact of sex on model predicted weigh Z-score trajectories for juvenile rheumatic disease groups and matched controls.

Figure 12: The impact of pubertal stage at diagnosis of juvenile disease on model predicted height trajectories for juvenile rheumatic disease groups and matched controls

Figure 13: The impact of pubertal stage at diagnosis of juvenile disease on model predicted weight Z-score trajectories for juvenile rheumatic disease groups and matched controls

Figure 14: The impact of pubertal stage at diagnosis of juvenile disease on model predicted weight trajectories for juvenile rheumatic disease groups and matched controls

**TABLES:**

Table 1: SNOMED codes for defining Juvenile Idiopathic Arthritis. Note that where adult rheumatic codes have been included (e.g. Rheumatoid arthritis) we also specified that the individual was under 18 at the point at which the code was observed.

| **Medcode ID** | **Term** | **Original Read Code** | **Cleansed Read Code** | **Snomed CT Concept Id** | **Snomed CT Description Id** |
| --- | --- | --- | --- | --- | --- |
| 309829012 | Other juvenile arthritis | N045 | N045.00 | 239796000 | 2.7401E+13 |
| 312523013 | [X]Other juvenile arthritis | Nyu15 | Nyu1500 | 239796000 | 4.1135E+14 |
| 483981000006116 | Ankylosis of multiple joints | N0859 | N085900 | 90242006 | 4.8398E+14 |
| 178449010 | Ankylosis of joint | N085 | N085.00 | 111227009 | 178449010 |
| 310553013 | Ankylosis of the subtalar joint | N085R | N085R00 | 202319005 | 310553013 |
| 310557014 | Ankylosis of joint NOS | N085z | N085z00 | 111227009 | 4.8395E+14 |
| 310539013 | Ankylosis of the elbow joint | N085C | N085C00 | 202307002 | 310539013 |
| 310550011 | Ankylosis of other pelvic joint | N085N | N085N00 | 267934008 | 4.8401E+14 |
| 310554019 | Ankylosis of other tarsal joint | N085S | N085S00 | 267936005 | 4.8402E+14 |
| 310543012 | Ankylosis of the wrist joint | N085F | N085F00 | 202310009 | 310543012 |
| 483971000006119 | Ankylosis of MTP joint | N085T | N085T00 | 202321000 | 4.8397E+14 |
| 310556017 | Ankylosis of toe joint | N085U | N085U00 | 202322007 | 310556017 |
| 359848019 | Ankylosis of spine NOS | N14z-1 | N14z.11 | 47049007 | 4.8404E+14 |
| 890391000006115 | Ankylosis - shoulder joint | N0851-99 | N085199 | 202298000 | 8.9039E+14 |
| 890451000006119 | Ankylosis - ankle/foot | N0857-99 | N085799 | 267936005 | 8.9045E+14 |
| 483991000006118 | Ankylosis of other CMC joint | N085H | N085H00 | 202310009 | 4.8399E+14 |
| 484071000006116 | Ankylosis of the ankle joint | N085Q | N085Q00 | 202318002 | 4.8407E+14 |
| 484121000006116 | Ankylosis of the shoulder joint | N085A | N085A00 | 202298000 | 4.8412E+14 |
| 890411000006115 | Ankylosis - wrist joint | N0853-99 | N085399 | 267933002 | 8.9041E+14 |
| 890431000006114 | Ankylosis - hip joint | N0855-99 | N085599 | 267934008 | 8.9043E+14 |
| 890441000006116 | Ankylosis - knee joint | N0856-99 | N085699 | 267935009 | 8.9044E+14 |
| 484031000006119 | Ankylosis of PIP joint | N085K | N085K00 | 202314000 | 4.8403E+14 |
| 484111000006112 | Ankylosis of the knee joint | N085P | N085P00 | 202317007 | 4.8411E+14 |
| 484051000006114 | Ankylosis of teeth | J016 | J016.00 | 14901003 | 4.8405E+14 |
| 484141000006111 | Ankylosis of the superior radio-ulnar joint | N085D | N085D00 | 202308007 | 4.8414E+14 |
| 890371000006116 | Ankylosis - multiple joint | N0849-98 | N084998 | 202264009 | 8.9037E+14 |
| 890401000006118 | Ankylosis - elbow joint | N0852-99 | N085299 | 267932007 | 8.904E+14 |
| 310538017 | Ankylosis of other joint of the shoulder girdle | N085B | N085B00 | 302154007 | 4.84E+14 |
| 483961000006114 | Ankylosis of MCP joint | N085J | N085J00 | 202313006 | 4.8396E+14 |
| 484171000006115 | Ankylosis/instability of cervical,thoracic or lumbar spine | N148 | N148.00 | 47049007 | 4.8417E+14 |
| 890461000006117 | Ankylosis - other joint | N0858-99 | N085899 | 7.0154E+14 | 8.9046E+14 |
| 890421000006111 | Ankylosis - hand joint | N0854-99 | N085499 | 56923001 | 8.9042E+14 |
| 483931000006117 | Ankylosis of DIP joint | N085L | N085L00 | 202315004 | 4.8393E+14 |
| 484061000006111 | Ankylosis of the 1st CMC joint | N085G | N085G00 | 202311008 | 4.8406E+14 |
| 484091000006115 | Ankylosis of the hip joint | N085M | N085M00 | 24273009 | 4.8409E+14 |
| 484101000006114 | Ankylosis of the inferior radio-ulnar joint | N085E | N085E00 | 202309004 | 4.841E+14 |
| 359321011 | Distal interphalangeal psoriatic arthropathy | M1601 | M160100 | 239812005 | 359321011 |
| 312521010 | [X]Other psoriatic arthropathies | Nyu13 | Nyu1300 | 33339001 | 4.1308E+14 |
| 312518013 | [X]Other seropositive rheumatoid arthritis | Nyu11 | Nyu1100 | 239791005 | 4.1353E+14 |
| 312520011 | [X]Other specified rheumatoid arthritis | Nyu12 | Nyu1200 | 69896004 | 4.1632E+14 |
| 312534011 | [X]Seropositive rheumatoid arthritis, unspecified | Nyu1G | Nyu1G00 | 239791005 | 4.2651E+14 |
| 125937016 | Acute polyarticular juvenile rheumatoid arthritis | N0431 | N043100 | 75822003 | 125937016 |
| 371261000000111 | Delivery of rehabilitation for rheumatoid arthritis | 7P203 | 7P20300 | 2.3104E+14 | 3.7126E+14 |
| 1785241000006114 | Except rheumatoid arthritis qual indicator: informed dissent | 9hR1 | 9hR1.00 | 8.0598E+14 | 1785241000006114 |
| 1785231000006116 | Except rheumatoid arthritis quality indicator: pt unsuitable | 9hR0 | 9hR0.00 | 8.0596E+14 | 1785231000006116 |
| 311496011 | Fibrosing alveolitis associated with rheumatoid arthritis | N04y0-2 | N04y012 | 10713006 | 7.0951E+14 |
| 309816012 | Flare of rheumatoid arthritis | N040T | N040T00 | 201791009 | 309816012 |
| 309828016 | Juvenile rheumatoid arthritis NOS | N043z | N043z00 | 410795001 | 7.5545E+14 |
| 2472447010 | Juvenile rheumatoid arthritis | N0455 | N045500 | 410795001 | 2472447010 |
| 755441000006119 | Juvenile rheumatoid arthritis - Still's disease | N043 | N043.00 | 410795001 | 7.5544E+14 |
| 297641010 | Myopathy due to rheumatoid arthritis | F3964 | F396400 | 193250002 | 297641010 |
| 309827014 | Monarticular juvenile rheumatoid arthritis | N0433 | N043300 | 201799006 | 309827014 |
| 123542016 | Pauciarticular juvenile rheumatoid arthritis | N0432 | N043200 | 74391003 | 123542016 |
| 297544012 | Polyneuropathy in rheumatoid arthritis | F3712 | F371200 | 193180002 | 297544012 |
| 1785221000006119 | Exception reporting: rheumatoid arthritis quality indicators | 9hR | 9hR..00 | 8.0594E+14 | 1785221000006119 |
| 251794010 | H/O: rheumatoid arthritis | 14G1 | 14G1.00 | 161567008 | 251794010 |
| 309788014 | Other rheumatoid arthritis of spine | N0401 | N040100 | 9631008 | 1.6901E+13 |
| 451461014 | Seropositive errosive rheumatoid arthritis | N047 | N047.00 | 308143008 | 451461014 |
| 359292012 | Seronegative rheumatoid arthritis | N040P | N040P00 | 239792003 | 359292012 |
| 149911000006117 | Seropositive rheumatoid arthritis, unspecified | N04X | N04X.00 | 239791005 | 1.4991E+14 |
| 312529012 | [X]Other specified arthritis | Nyu1B | Nyu1B00 | 3723001 | 4.1406E+14 |
| 250085017 | FH: Rheumatoid arthritis | 12I1 | 12I1.00 | 160413008 | 250085017 |
| 309789018 | Rheumatoid arthritis of shoulder | N0402 | N040200 | 201766009 | 309789018 |
| 2159844013 | Rheumatoid arthritis particle agglutination test | 43b9 | 43b9.00 | 1010071000000108 | 2582321000000111 |
| 162131000006111 | Rheumatoid arthritis of PIP joint of finger | N0409 | N040900 | 201773004 | 1.6213E+14 |
| 116082011 | Rheumatoid arthritis | N040 | N040.00 | 69896004 | 116082011 |
| 1875591000006110 | Rheumatoid arthritis monitoring invitation first letter | EMISNQRH20 | | 1875591000006106 | 1875591000006110 |
| 2196791000000119 | Rheumatoid arthritis annual review | 66HB0 | 66HB000 | 8.4726E+14 | 2196791000000119 |
| 2275501000000119 | Rheumatoid arthritis monitoring invitation | 9mM | 9mM..00 | 8.8232E+14 | 2275501000000119 |
| 309794018 | Rheumatoid arthritis of wrist | N0407 | N040700 | 201771002 | 309794018 |
| 309800010 | Rheumatoid arthritis of knee | N040D | N040D00 | 201777003 | 309800010 |
| 309805017 | Rheumatoid arthritis of other tarsal joint | N040J | N040J00 | 429192004 | 1.6212E+14 |
| 309804018 | Rheumatoid arthritis of talonavicular joint | N040H | N040H00 | 201781003 | 309804018 |
| 309798015 | Rheumatoid arthritis of hip | N040B | N040B00 | 201775006 | 309798015 |
| 309792019 | Rheumatoid arthritis of elbow | N0405 | N040500 | 201769002 | 309792019 |
| 162041000006117 | Rheumatoid arthritis of DIP joint of finger | N040A | N040A00 | 201774005 | 1.6204E+14 |
| 162051000006115 | Rheumatoid arthritis of distal radio-ulnar joint | N0406 | N040600 | 201770001 | 1.6205E+14 |
| 309787016 | Rheumatoid arthritis of cervical spine | N0400 | N040000 | 201764007 | 309787016 |
| 309790010 | Rheumatoid arthritis of sternoclavicular joint | N0403 | N040300 | 201767000 | 309790010 |
| 309791014 | Rheumatoid arthritis of acromioclavicular joint | N0404 | N040400 | 201768005 | 309791014 |
| 426510015 | Rheumatoid arthritis - multiple joint | N040S | N040S00 | 287006005 | 426510015 |
| 162101000006115 | Rheumatoid arthritis of lesser MTP joint | N040L | N040L00 | 201784006 | 1.621E+14 |
| 162211000006111 | Rheumatoid arthritis screen | 68F1 | 68F1.00 | 1016811000000108 | 1.6221E+14 |
| 168761000006118 | Rheumatoid arthritis of 1st MTP joint | N040K | N040K00 | 201783000 | 1.6876E+14 |
| 889731000006111 | Rheumatoid arthritis NOS | N040-90 | N040.90 | 69896004 | 8.8973E+14 |
| 162191000006110 | Rheumatoid arthritis of tibio-fibular joint | N040E | N040E00 | 201778008 | 1.6219E+14 |
| 162081000006111 | Rheumatoid arthritis of IP joint of toe | N040M | N040M00 | 201785007 | 1.6208E+14 |
| 162111000006117 | Rheumatoid arthritis of MCP joint | N0408 | N040800 | 201772009 | 1.6211E+14 |
| 162221000006115 | Rheumatoid arthritis screening test | 43c6 | 43c6.00 | 1016811000000108 | 2565831000000112 |
| 168751000006115 | Rheumatoid arthritis and other inflammatory polyarthropathy | N04 | N04..00 | 69896004 | 1.6875E+14 |
| 309802019 | Rheumatoid arthritis of ankle | N040F | N040F00 | 201779000 | 309802019 |
| 309803012 | Rheumatoid arthritis of subtalar joint | N040G | N040G00 | 201780002 | 309803012 |
| 909191000006119 | [RFC] Rheumatoid arthritis | HNGP005 |  | 9.0919E+14 | 9.0919E+14 |
| 2275751000000111 | Rheumatoid arthritis monitoring invitation third letter | 9mM2 | 9mM2.00 | 8.8244E+14 | 2275751000000111 |
| 1875611000006116 | Rheumatoid arthritis monitoring invitation third letter | EMISNQRH22 | | 1875611000006100 | 1875611000006116 |
| 1875581000006112 | Rheumatoid arthritis monitoring verbal invitation | EMISNQRH19 | | 1875581000006108 | 1875581000006112 |
| 1875601000006119 | Rheumatoid arthritis monitoring invitation second letter | EMISNQRH21 | | 1875601000006103 | 1875601000006119 |
| 1880371000006117 | Rheumatoid arthritis resolved | EMISNQRH23 | | 1880371000006101 | 1880371000006117 |
| 1875571000006114 | Rheumatoid arthritis monitoring telephone invitation | EMISNQRH18 | | 1875571000006105 | 1875571000006114 |
| 2275831000000110 | Rheumatoid arthritis monitoring telephone invitation | 9mM4 | 9mM4.00 | 8.8248E+14 | 2275831000000110 |
| 2275711000000112 | Rheumatoid arthritis monitoring invitation second letter | 9mM1 | 9mM1.00 | 8.8242E+14 | 2275711000000112 |
| 2275791000000115 | Rheumatoid arthritis monitoring verbal invitation | 9mM3 | 9mM3.00 | 8.8246E+14 | 2275791000000115 |
| 162141000006118 | Rheumatoid arthritis of sacro-iliac joint | N040C | N040C00 | 201776007 | 1.6214E+14 |
| 424981000006114 | [X]Rheumatoid arthritis+involvement/other organs or systems | Nyu10 | Nyu1000 | 239793008 | 4.2498E+14 |
| 1875561000006119 | Rheumatoid arthritis monitoring invitation | EMISNQRH17 | | 1875561000006103 | 1875561000006119 |
| 2275671000000111 | Rheumatoid arthritis monitoring invitation first letter | 9mM0 | 9mM0.00 | 8.824E+14 | 2275671000000111 |
| 20587019 | Acute arthritis | N06zA | N06zA00 | 11939005 | 20587019 |
| 359168010 | Ankle arthritis NOS | N06z7-1 | N06z711 | 239874001 | 4.8369E+14 |
| 310552015 | Ankle joint ankylosis | N0857-1 | N085711 | 202318002 | 310552015 |
| 16833013 | Ankylosing spondylitis | N100 | N100.00 | 9631008 | 16833013 |
| 484889013 | Ankylosing vertebral hyperostosis | N117 | N117.00 | 31487001 | 484889013 |
| 1488690018 | FH: Ankylosing spondylitis | 12I7 | 12I7.00 | 394990003 | 1488690018 |
| 2325761000000118 | Bath Ankylosing Spondylitis Functional Index | 388p0 | 388p000 | 9.0431E+14 | 2325761000000118 |
| 2326221000000110 | Bath Ankylosing Spondylitis Metrology Index | 38QL | 38QL.00 | 9.0451E+14 | 2326221000000110 |
| 1743391000006117 | Ankylosing vertebral hyperostosis | N117-z376 |  | 1743391000006101 | 1743391000006117 |
| 497061000006119 | Atlanto-axial ankylosis | N1481 | N148100 | 202813002 | 4.9706E+14 |
| 311288015 | Atlanto-occipital instability | N1487 | N148700 | 202819003 | 311288015 |
| 311281014 | Atlanto-occipital ankylosis | N1480 | N148000 | 202812007 | 311281014 |
| 497071000006114 | Atlanto-axial instability | N1488 | N148800 | 202820009 | 4.9707E+14 |
| 1898101000006110 | Axial spondyloarthritis | EMISNQAX2 |  | 1898101000006106 | 1898101000006110 |
| 2360741000000115 | Axial spondyloarthritis | N11F | N11F.00 | 723116002 | 3334645019 |
| 2692996011 | BASDAI - Bath ankylosing spondylitis disease activity index | 388p | 388p.00 | 429712009 | 2692996011 |
| 1786505011 | Caplan's syndrome | N04y0-1 | N04y011 | 398640008 | 1786505011 |
| 311283012 | Cervical spine ankylosis | N1482 | N148200 | 202814008 | 311283012 |
| 311290019 | Cervical spine instability | N1489 | N148900 | 202821008 | 311290019 |
| 544821000006112 | Cervico-thoracic instability | N148A | N148A00 | 202822001 | 5.4482E+14 |
| 544811000006116 | Cervico-thoracic ankylosis | N1483 | N148300 | 202815009 | 5.4481E+14 |
| 59922014 | Chronic arthritis | N06zB | N06zB00 | 35908007 | 59922014 |
| 359172014 | Elbow arthritis NOS | N06z2-1 | N06z211 | 439656005 | 6.3739E+14 |
| 95067016 | Felty's syndrome | N041 | N041.00 | 57160007 | 95067016 |
| 359167017 | Foot arthritis NOS | N06z7-2 | N06z712 | 448394006 | 7.6424E+14 |
| 310159018 | Generalised arthritis | N065A | N065A00 | 202031002 | 310159018 |
| 815501000006110 | Hand arthritis NOS | N06z4-1 | N06z411 | 448589005 | 8.155E+14 |
| 359170018 | Hip arthritis NOS | N06z5-1 | N06z511 | 68449006 | 8.2375E+14 |
| 481879010 | Hip joint ankylosis | N0855-1 | N085511 | 24273009 | 481879010 |
| 310551010 | Knee joint ankylosis | N0856-1 | N085611 | 202317007 | 310551010 |
| 108781000006112 | Temporomandibular joint ankylosis | J0460 | J046000 | 50603008 | 1.0878E+14 |
| 310544018 | Wrist joint ankylosis | N0853-1 | N085311 | 202310009 | 310544018 |
| 310540010 | Elbow joint ankylosis | N0852-1 | N085211 | 202307002 | 310540010 |
| 312611014 | [X]Other specified inflammatory spondylopathies | Nyu61 | Nyu6100 | 202649003 | 4.156E+14 |
| 312615017 | [X]Other specified spondylopathies | Nyu65 | Nyu6500 | 4.1483E+14 | 4.1639E+14 |
| 311043013 | Inflammatory spondylopathies in diseases EC | N10y0 | N10y000 | 202652006 | 311043013 |
| 312610010 | [X]Other infective spondylopathies | Nyu60 | Nyu6000 | 721273004 | 4.1114E+14 |
| 311044019 | Other inflammatory spondylopathies NOS | N10yz | N10yz00 | 202649003 | 2.8481E+13 |
| 311040011 | Inflammatory spondylopathies | N10 | N10..00 | 202649003 | 311040011 |
| 311042015 | Other inflammatory spondylopathies | N10y | N10y.00 | 202649003 | 2.8471E+13 |
| 890821000006113 | Inflam. spondylopathies NOS | N10z-99 | N10z.99 | 6.8523E+14 | 8.9082E+14 |
| 310518018 | Joint ankylosis of unspecified site | N0850 | N085000 | 111227009 | 7.5789E+14 |
| 400222018 | Joint ankylosis of the upper arm | N0852 | N085200 | 267932007 | 400222018 |
| 310519014 | Joint ankylosis of the shoulder region | N0851 | N085100 | 202298000 | 310519014 |
| 310537010 | Joint ankylosis of other specified site | N0858 | N085800 | 111227009 | 7.5781E+14 |
| 400226015 | Joint ankylosis of the ankle and foot | N0857 | N085700 | 267936005 | 400226015 |
| 400223011 | Joint ankylosis of the forearm | N0853 | N085300 | 202310009 | 7.5783E+14 |
| 400225016 | Joint ankylosis of the lower leg | N0856 | N085600 | 267935009 | 400225016 |
| 400224017 | Joint ankylosis of the pelvic region and thigh | N0855 | N085500 | 267934008 | 400224017 |
| 757841000006114 | Joint ankylosis of the hand | N0854 | N085400 | 56923001 | 7.5784E+14 |
| 309836013 | Juvenile arthritis in ulcerative colitis | N0454 | N045400 | 201807008 | 309836013 |
| 309833017 | Juvenile arthritis in Crohn's disease | N0453 | N045300 | 201805000 | 309833017 |
| 359310019 | Juvenile arthritis in psoriasis | N0452 | N045200 | 239803008 | 359310019 |
| 359312010 | Juvenile ankylosing spondylitis | N0450 | N045000 | 239805001 | 359312010 |
| 309824019 | Juvenile rheumatoid arthropathy unspecified | N0430 | N043000 | 410793008 | 7.5546E+14 |
| 2472449013 | Juvenile seronegative polyarthritis | N0451 | N045100 | 410797009 | 7.5547E+14 |
| 359169019 | Knee arthritis NOS | N06z6-1 | N06z611 | 371081002 | 7.537E+14 |
| 311287013 | Lumbar spine ankylosis | N1486 | N148600 | 202818006 | 311287013 |
| 311293017 | Lumbar spine instability | N148C | N148C00 | 202824000 | 311293017 |
| 1235278014 | Lumbosacral ankylosis | N1460 | N146000 | 87823004 | 1235278014 |
| 299135012 | Malleus ankylosis | F5520 | F552000 | 194333008 | 299135012 |
| 718001000006112 | Marie - Strumpell spondylitis | N100-1 | N100.11 | 9631008 | 7.18E+14 |
| 255925015 | O/E-hands-rheumatoid spindling | 2G27 | 2G27.00 | 164456005 | 255925015 |
| 253940015 | O/E - ankyl.spondyl.chest def. | 2377 | 2377 | 162930007 | 253940015 |
| 299136013 | Ossicle ankylosis (excluding malleus) | F5521 | F552100 | 194334002 | 299136013 |
| 16911000006114 | Other rheumatoid arthropathy + visceral/systemic involvement | N042 | N042.00 | 239793008 | 1.6911E+13 |
| 502690012 | Pauciarticular onset juvenile chronic arthritis | N0456 | N045600 | 74391003 | 502690012 |
| 311398011 | Periarthritis NOS | N21z1 | N21z100 | 50921008 | 2.3385E+14 |
| 178460018 | Periarthritis of wrist | N2142 | N214200 | 111242007 | 178460018 |
| 1786700015 | Periarthritis of shoulder | N2120 | N212000 | 399114005 | 1786700015 |
| 221661000006114 | Polyarthritis | N065z-1 | N065z11 | 417373000 | 2.2166E+14 |
| 55628018 | Psoriatic arthritis | M160-1 | M160.11 | 33339001 | 55628018 |
| 55627011 | Psoriatic arthropathy | M160 | M160.00 | 33339001 | 55627011 |
| 308730016 | Psoriatic arthropathy NOS | M160z | M160z00 | 33339001 | 1.9893E+14 |
| 198881000006116 | Psoriasis spondylitica | M1600 | M160000 | 200956002 | 1.9888E+14 |
| 1756301000006119 | Remitting seronegative symmetrical synovitis pitting oedema | N04y3 | N04y300 | 445345005 | 1756301000006119 |
| 1746141000006118 | Remitting seronegative symmetrical synovitis with pitting oedema | EMISNQRE122 | | 1746141000006102 | 1746141000006118 |
| 39771016 | Rheumatic carditis | N0420 | N042000 | 23685000 | 39771016 |
| 162231000006117 | Rheumatoid arthropathy + visceral/systemic involvement NOS | N042z | N042z00 | 239793008 | 1.6223E+14 |
| 309812014 | Rheumatoid bursitis | N040Q | N040Q00 | 201788009 | 309812014 |
| 48361011 | Rheumatoid carditis | G5yA | G5yA.00 | 28880005 | 48361011 |
| 1778239014 | Rheumatoid lung disease | N0421 | N042100 | 398726004 | 1778239014 |
| 162301000006117 | Rheumatoid lung | H570 | H570.00 | 398726004 | 1.623E+14 |
| 1786545019 | Rheumatoid lung | N04y0 | N04y000 | 398726004 | 1786545019 |
| 300221014 | Rheumatoid myocarditis | G5y8 | G5y8.00 | 195136004 | 300221014 |
| 311041010 | Sacroiliitis NEC | N102 | N102.00 | 55146009 | 1.5986E+14 |
| 311267012 | Sacral ankylosis NOS | N1462 | N146200 | 16065006 | 1.5957E+14 |
| 1221279016 | Sacroiliac ankylosis | N1461 | N146100 | 16065006 | 1221279016 |
| 311045018 | Spondylitis NOS | N10z | N10z.00 | 84172003 | 1.2992E+14 |
| 17967011 | Spinal enthesopathy | N101 | N101.00 | 10317009 | 17967011 |
| 219271000000117 | Sero negative arthritis | N04y1 | N04y100 | 399112009 | 2.1927E+14 |
| 215551000000114 | Sero negative polyarthritis | N04y1-1 | N04y111 | 399112009 | 2.1555E+14 |
| 140551000006117 | Shoulder arthritis NOS | N06z1-1 | N06z111 | 443798008 | 1.4055E+14 |
| 559621000000119 | Suspected inflammatory arthritis | 1JG | 1JG..00 | 431956005 | 2768203016 |
| 311285017 | Thoracic spine ankylosis | N1484 | N148400 | 202816005 | 311285017 |
| 311292010 | Thoracic spine instability | N148B | N148B00 | 202823006 | 311292010 |
| 106931000006114 | Thoraco-lumbar ankylosis | N1485 | N148500 | 202817001 | 1.0693E+14 |
| 1464018 | Unspecified monoarthritis | N066 | N066.00 | 699462004 | 7.7391E+13 |
| 310170013 | Unspecified monoarthritis of the hand | N0664 | N066400 | 698475006 | 7.7441E+13 |
| 310172017 | Unspecified monoarthritis of the lower leg | N0666 | N066600 | 699462004 | 7.7451E+13 |
| 310169012 | Unspecified monoarthritis of the forearm | N0663 | N066300 | 699462004 | 7.7431E+13 |
| 310167014 | Unspecified monoarthritis of the shoulder region | N0661 | N066100 | 699462004 | 7.7471E+13 |
| 310174016 | Unspecified monoarthritis of other specified site | N0668 | N066800 | 699462004 | 7.7411E+13 |
| 310171012 | Unspecified monoarthritis of the pelvic region and thigh | N0665 | N066500 | 699462004 | 7.7461E+13 |
| 310175015 | Unspecified monoarthritis NOS | N066z | N066z00 | 699462004 | 7.7401E+13 |
| 310168016 | Unspecified monoarthritis of the upper arm | N0662 | N066200 | 699462004 | 7.7481E+13 |
| 310166017 | Unspecified monoarthritis of unspecified site | N0660 | N066000 | 699462004 | 7.7491E+13 |
| 310173010 | Unspecified monoarthritis of the ankle and foot | N0667 | N066700 | 699462004 | 7.7421E+13 |
| 405142017 | Unspecified polyarthropathy or polyarthritis | N065 | N065.00 | 36186002 | 7.8151E+13 |
| 400192018 | Unspecified polyarthropathy or polyarthritis NOS | N065z | N065z00 | 36186002 | 7.8161E+13 |
| 359171019 | Wrist arthritis NOS | N06z3-1 | N06z311 | 1.771E+12 | 5.4521E+13 |

Table 2: SNOMED codes for defining Juvenile-onset Systemic Lupus Erythematosus. Note that where adult lupus codes have been included, we also specified that the individual was under 18 at the point at which the code was observed.

| **Medcode ID** | **Term** | **Original Read Code** | **Cleansed Read Code** | **Snomed CT Concept Id** | **Snomed CT Description Id** |
| --- | --- | --- | --- | --- | --- |
| 92209015 | Disseminated lupus erythematosus | N0000 | N000000 | 55464009 | 92209015 |
| 92208011 | Systemic lupus erythematosus | N000 | N000.00 | 55464009 | 92208011 |
| 297542011 | Polyneuropathy in disseminated lupus erythematosus | F3710 | F371000 | 193178008 | 297542011 |
| 301721015 | Lung disease with systemic lupus erythematosus | H57y4 | H57y400 | 196138005 | 301721015 |
| 308701011 | Discoid lupus erythematosus | M1541 | M154100 | 200938002 | 308701011 |
| 309408013 | Systemic lupus erythematosus NOS | N000z | N000z00 | 55464009 | 1.1424E+14 |
| 312579010 | [X]Other forms of systemic lupus erythematosus | Nyu43 | Nyu4300 | 55464009 | 4.1051E+14 |
| 512234017 | Cerebral lupus | N0006 | N000600 | 95644001 | 512234017 |
| 359447015 | Subacute cutaneous lupus erythematosus | M1547 | M154700 | 239891002 | 359447015 |
| 453243010 | Systemic lupus erythematosus with pericarditis | N0004 | N000400 | 309762007 | 453243010 |
| 309383019 | [X]Other local lupus erythematosus | Myu78 | Myu7800 | 7119001 | 4.1149E+14 |
| 114251000006118 | Systemic lupus erythematosus with organ or sys involv | N0003 | N000300 | 239887007 | 1.1425E+14 |
| 677561000006119 | Nephrotic syndrome in systemic lupus erythematosus | K01x4 | K01x400 | 68815009 | 6.7756E+14 |
| 660871000006118 | Eyelid discoid lupus erythematosus | F4D33 | F4D3300 | 79291003 | 6.6087E+14 |
| 25612013 | Lupus erythematosus profundus | M1544 | M154400 | 15084002 | 25612013 |
| 308706018 | Lupus erythematosus tumidus | M1545 | M154500 | 200941006 | 308706018 |
| 308697013 | Lupus erythematosus | M154 | M154.00 | 200936003 | 308697013 |
| 308709013 | Lupus erythematosus NOS | M154z | M154z00 | 200936003 | 7.3214E+14 |
| 308700012 | Lupus erythematosus chronicus | M1540 | M154000 | 200937007 | 308700012 |
| 308704015 | Lupus erythematosus migrans | M1542 | M154200 | 200939005 | 308704015 |
| 308705019 | Lupus erythematosus nodularis | M1543 | M154300 | 200940007 | 308705019 |
| 308707010 | Lupus erythematosus unguium mutilans | M1546 | M154600 | 200942004 | 308707010 |
| 114310015 | Lupus nephritis | K01x4-1 | K01x411 | 68815009 | 114310015 |

Table 3: SNOMED codes for defining Juvenile Dermatomyositis. Note that where adult codes have been included, we also specified that the individual was under 18 at the point at which the code was observed.

| **Medcode ID** | **Term** | **Original Read Code** | **Cleansed Read Code** | **Snomed CT Concept Id** | **Snomed CT Description Id** |
| --- | --- | --- | --- | --- | --- |
| 1776211019 | Dermatomyositis | N003 | N003.00 | 396230008 | 1776211019 |
| 312591018 | [X]Dermatopolymyositis, unspecified | Nyu4E | Nyu4E00 | 419921000000105 | 939011000000110 |
| 614691000006113 | Dermatopolymyositis, unspecified | N003X | N003X00 | 419921000000105 | 614691000006113 |
| 143230018 | Poikilodermatomyositis | N003-1 | N003.11 | 396230008 | 220521000006113 |
| 1784692011 | Juvenile dermatomyositis | N0030 | N003000 | 1212005 | 1784692011 |
| 312580013 | [X]Other dermatomyositis | Nyu44 | Nyu4400 | 396230008 | 408771000006110 |
| 301719013 | Lung disease with polymyositis | H57y1 | H57y100 | 196136009 | 301719013 |
| 52463013 | Polymyositis | N004 | N004.00 | 31384009 | 52463013 |

Table 4: SNOMED codes for defining height measurements

| **Medcode ID** | **Term** | **Original Read Code** | **Cleansed Read Code** | **Snomed CT Concept Id** | **Snomed CT Description Id** |
| --- | --- | --- | --- | --- | --- |
| 253672015 | O/E -height within 10% average | 2293 | 2293.00 | 162758008 | 253672015 |
| 284561000006119 | O/E - height 10-20% < average | 2292 | 2292.00 | 162757003 | 284561000006119 |
| 253675018 | O/E - loss of height | 2296 | 2296.00 | 162761009 | 253675018 |
| 923831000006115 | O/E - height | PCNQOE1 |  | 923831000006104 | 923831000006115 |
| 253676017 | O/E - height NOS | 229Z | 229Z.00 | 162755006 | 284571000006114 |
| 253673013 | O/E-height 10-20% over average | 2294 | 2294.00 | 162759000 | 253673013 |
| 253669010 | O/E - height | 229 | 229..00 | 162755006 | 253669010 |
| 271181000006113 | O/E-height > 20% below average | 2291 | 2291.00 | 162756007 | 271181000006113 |
| 272151000006114 | O/E -height > 20% over average | 2295 | 2295.00 | 162760005 | 272151000006114 |

Table 5: SNOMED codes for defining weight measurements

| **Medcode ID** | **Term** | **Original Read Code** | **Cleansed Read Code** | **Snomed CT Concept Id** | **Snomed CT Description Id** |
| --- | --- | --- | --- | --- | --- |
| 253677014 | O/E - weight | 22A | 22A..00 | 162763007 | 253677014 |
| 402435016 | O/E - weight 10-20% over ideal | 22A4 | 22A4.00 | 268915006 | 402435016 |
| 253688015 | O/E - weight NOS | 22AZ | 22AZ.00 | 162763007 | 272581000006112 |
| 253680010 | O/E - weight within 10% ideal | 22A3 | 22A3.00 | 162766004 | 253680010 |
| 1780188013 | O/E - weight-bearing | 29N | 29N..00 | 400981002 | 1780188013 |
| 272551000006116 | O/E - weight > 20% below ideal | 22A1 | 22A1.00 | 162764001 | 272551000006116 |
| 217781000000116 | O/E - weight > 20% over ideal | 22A5 | 22A5.00 | 268916007 | 217781000000116 |
| 923851000006110 | O/E - weight | PCNQOE3 |  | 923851000006106 | 923851000006110 |

**Results of analyses using absolute height and weight measurements**

Table 6: Linear mixed model results for height measurements in the JIA cohort

|  | **Whole Cohort** | | | | **Females** | | | | **Males** | | | |
| --- | --- | --- | --- | --- | --- | --- | --- | --- | --- | --- | --- | --- |
|  | Estimate | 95% CI | | p-value | Estimate | 95% CI | | p-value | Estimate | 95% CI | | p-value |
| **Intercept** | 127.170 | 126.277 | 128.069 | <0.001 | 127.093 | 126.071 | 128.129 | <0.001 | 115.036 | 113.856 | 116.227 | <0.001 |
| **bs(Time_since_diagnosis, 3)1** | 22.990 | 22.050 | 23.929 | <0.001 | 20.360 | 19.268 | 21.449 | <0.001 | 30.905 | 29.240 | 32.566 | <0.001 |
| **bs(Time_since_diagnosis, 3)2** | 45.299 | 43.812 | 46.787 | <0.001 | 33.725 | 32.061 | 35.390 | <0.001 | 67.194 | 64.417 | 69.971 | <0.001 |
| **bs(Time_since_diagnosis, 3)3** | 4.289 | 2.263 | 6.316 | <0.001 | -13.207 | -15.432 | -10.983 | <0.001 | 51.711 | 47.678 | 55.758 | <0.001 |
| **JIA** | -1.754 | -2.546 | -0.962 | <0.001 | -2.333 | -3.273 | -1.394 | <0.001 | -0.419 | -1.788 | 0.950 | 0.548 |
| **Pubertal_Stage = Adolescent** | 33.745 | 32.834 | 34.650 | <0.001 | 26.452 | 25.261 | 27.628 | <0.001 | 47.451 | 45.767 | 49.120 | <0.001 |
| **Pubertal_Stage = Peri-pubertal** | 27.656 | 26.925 | 28.386 | <0.001 | 22.870 | 21.870 | 23.862 | <0.001 | 33.272 | 32.124 | 34.417 | <0.001 |
| **Female** | -7.417 | -7.948 | -6.886 | <0.001 |  |  |  |  |  |  |  |  |
| **Ethnicity = Asian** | -2.921 | -3.915 | -1.927 | <0.001 | -3.688 | -4.831 | -2.546 | <0.001 | -2.221 | -4.026 | -0.416 | 0.016 |
| **Ethnicity = Black** | 0.879 | -0.682 | 2.439 | 0.270 | 0.463 | -1.317 | 2.244 | 0.610 | 0.840 | -1.998 | 3.678 | 0.562 |
| **Ethnicity = Other** | -0.279 | -1.116 | 0.559 | 0.514 | -0.101 | -1.003 | 0.800 | 0.826 | -0.412 | -2.125 | 1.301 | 0.638 |
| **bs(Time_since_diagnosis, 3)1 * JIA** | 1.960 | 0.646 | 3.276 | 0.003 | 1.650 | 0.148 | 3.154 | 0.030 | 0.990 | -1.317 | 3.299 | 0.400 |
| **bs(Time_since_diagnosis, 3)2 * JIA** | -0.229 | -2.585 | 2.125 | 0.849 | 1.900 | -0.725 | 4.521 | 0.155 | -2.000 | -6.259 | 2.255 | 0.357 |
| **bs(Time_since_diagnosis, 3)3 * JIA** | 4.915 | 1.545 | 8.285 | 0.004 | -0.013 | -3.767 | 3.741 | 0.995 | 7.873 | 1.639 | 14.101 | 0.013 |

Table 7: Linear mixed model results for height measurements in the JIA cohort, stratified by pubertal stage at diagnosis of rheumatic disease.

|  | **Pre-Pubertal** | | | | **Peri-Pubertal** | | | | **Adolescents** | | | |
| --- | --- | --- | --- | --- | --- | --- | --- | --- | --- | --- | --- | --- |
|  | Estimate | 95% CI | | p-value | Estimate | 95% CI | | p-value | Estimate | 95% CI | | p-value |
| **Intercept** | 109.798 | 108.597 | 111.005 | <0.001 | 156.031 | 155.143 | 156.928 | <0.001 | 175.779 | 175.094 | 176.466 | <0.001 |
| **bs(Time_since_diagnosis, 3)1** | 45.443 | 44.016 | 46.872 | <0.001 | 22.309 | 21.222 | 23.396 | <0.001 | 1.685 | 0.942 | 2.429 | <0.001 |
| **bs(Time_since_diagnosis, 3)2** | 82.053 | 80.482 | 83.628 | <0.001 | 21.498 | 20.285 | 22.713 | 0.029 | 2.310 | 1.481 | 3.138 | 0.144 |
| **bs(Time_since_diagnosis, 3)3** | 54.326 | 52.392 | 56.260 | <0.001 | 14.603 | 12.975 | 16.230 | <0.001 | 2.244 | 1.274 | 3.215 | 0.002 |
| **JIA** | -0.763 | -2.386 | 0.858 | 0.356 | -1.222 | -2.318 | -0.128 | 0.029 | -0.228 | -0.844 | 0.387 | 0.467 |
| **Female** | -7.335 | -8.170 | -6.504 | <0.001 | -9.820 | -10.511 | -9.133 | <0.001 | -12.152 | -12.819 | -11.484 | <0.001 |
| **Ethnicity = Asian** | -2.034 | -3.603 | -0.466 | 0.011 | -3.749 | -4.984 | -2.514 | <0.001 | -4.401 | -5.633 | -3.168 | <0.001 |
| **Ethnicity = Black** | 2.057 | -0.367 | 4.479 | 0.096 | -0.278 | -2.090 | 1.536 | 0.764 | -1.965 | -4.077 | 0.146 | 0.068 |
| **Ethnicity = Other** | 0.910 | -0.389 | 2.210 | 0.170 | -0.266 | -1.282 | 0.751 | 0.609 | -0.370 | -1.396 | 0.656 | 0.480 |
| **bs(Time_since_diagnosis, 3)1 * JIA** | -3.975 | -5.874 | -2.075 | <0.001 | -0.503 | -1.990 | 0.985 | 0.508 | 0.226 | -0.833 | 1.285 | 0.675 |
| **bs(Time_since_diagnosis, 3)2 * JIA** | 4.076 | 1.560 | 6.588 | 0.001 | 2.915 | 0.932 | 4.897 | 0.004 | -0.422 | -1.741 | 0.898 | 0.531 |
| **bs(Time_since_diagnosis, 3)3 * JIA** | 2.273 | -0.948 | 5.487 | 0.166 | -2.124 | -4.874 | 0.626 | 0.130 | -0.707 | -2.267 | 0.853 | 0.375 |

Table 8: Linear mixed model results for weight measurements in the JIA cohort

|  | **Whole Cohort** | | | | **Females** | | | | **Males** | | | |
| --- | --- | --- | --- | --- | --- | --- | --- | --- | --- | --- | --- | --- |
|  | Estimate | 95% CI | | p-value | Estimate | 95% CI | | p-value | Estimate | 95% CI | | p-value |
| **Intercept** | 20.501 | 19.758 | 21.244 | <0.001 | 20.903 | 20.146 | 21.659 | <0.001 | 16.232 | 15.111 | 17.354 | <0.001 |
| **bs(Time_since_diagnosis, 3)1** | 15.910 | 14.931 | 16.889 | <0.001 | 13.918 | 12.813 | 15.023 | <0.001 | 21.984 | 19.999 | 23.972 | <0.001 |
| **bs(Time_since_diagnosis, 3)2** | 46.250 | 44.725 | 47.776 | <0.001 | 40.382 | 38.690 | 42.075 | <0.001 | 60.107 | 56.885 | 63.321 | <0.001 |
| **bs(Time_since_diagnosis, 3)3** | 42.928 | 40.992 | 44.866 | <0.001 | 34.583 | 32.493 | 36.675 | <0.001 | 73.740 | 69.323 | 78.174 | <0.001 |
| **JIA** | -0.257 | -0.867 | 0.353 | 0.409 | -0.338 | -1.027 | 0.350 | 0.336 | -0.021 | -1.247 | 1.204 | 0.973 |
| **Pubertal_Stage = Adolescent** | 40.886 | 40.220 | 41.553 | <0.001 | 38.471 | 37.700 | 39.242 | <0.001 | 48.268 | 46.845 | 49.692 | <0.001 |
| **Pubertal_Stage = Peri-pubertal** | 29.233 | 28.588 | 29.879 | <0.001 | 28.378 | 27.598 | 29.160 | <0.001 | 29.719 | 28.526 | 30.908 | <0.001 |
| **Female** | -1.909 | -2.499 | -1.319 | <0.001 |  |  |  |  |  |  |  |  |
| **Ethnicity = Asian** | -2.756 | -3.837 | -1.675 | <0.001 | -3.302 | -4.607 | -1.997 | <0.001 | -2.246 | -4.165 | -0.327 | 0.022 |
| **Ethnicity = Black** | 2.234 | 0.533 | 3.935 | 0.010 | 2.546 | 0.481 | 4.612 | 0.016 | 1.873 | -1.107 | 4.853 | 0.218 |
| **Ethnicity = Other** | 0.542 | -0.368 | 1.453 | 0.243 | 0.651 | -0.387 | 1.689 | 0.219 | 0.153 | -1.664 | 1.968 | 0.869 |
| **bs(Time_since_diagnosis, 3)1 * JIA** | 1.508 | 0.143 | 2.874 | 0.030 | 0.926 | -0.628 | 2.481 | 0.243 | 1.917 | -0.775 | 4.608 | 0.163 |
| **bs(Time_since_diagnosis, 3)2 * JIA** | 2.167 | -0.302 | 4.635 | 0.085 | 3.696 | 0.914 | 6.476 | 0.009 | -0.689 | -5.615 | 4.244 | 0.784 |
| **bs(Time_since_diagnosis, 3)3 * JIA** | 2.991 | -0.345 | 6.328 | 0.079 | 3.089 | -0.592 | 6.772 | 0.100 | -1.788 | -8.822 | 5.242 | 0.618 |

Table 9: Linear mixed model results for weight measurements in the JIA cohort, stratified by pubertal stage at diagnosis of rheumatic disease.

|  | **Pre-Pubertal** | | | | **Peri-Pubertal** | | | | **Adolescents** | | | |
| --- | --- | --- | --- | --- | --- | --- | --- | --- | --- | --- | --- | --- |
|  | Estimate | 95% CI | | p-value | Estimate | 95% CI | | p-value | Estimate | 95% CI | | p-value |
| **Intercept** | 17.692 | 16.770 | 18.612 | <0.001 | 45.809 | 44.697 | 46.922 | <0.001 | 66.795 | 65.599 | 67.991 | <0.001 |
| **bs(Time_since_diagnosis, 3)1** | 17.990 | 16.323 | 19.664 | <0.001 | 22.341 | 20.815 | 23.869 | <0.001 | 3.658 | 2.607 | 4.708 | <0.001 |
| **bs(Time_since_diagnosis, 3)2** | 61.616 | 59.945 | 63.284 | <0.001 | 23.519 | 22.016 | 25.018 | <0.001 | 6.405 | 5.191 | 7.618 | <0.001 |
| **bs(Time_since_diagnosis, 3)3** | 54.930 | 52.915 | 56.958 | <0.001 | 29.984 | 28.022 | 31.948 | <0.001 | 11.603 | 10.150 | 13.056 | <0.001 |
| **JIA** | 0.160 | -0.890 | 1.212 | 0.765 | -0.068 | -1.259 | 1.124 | 0.912 | 0.921 | -0.003 | 1.846 | 0.051 |
| **Female** | -0.420 | -1.229 | 0.389 | 0.306 | -2.325 | -3.303 | -1.345 | <0.001 | -6.707 | -7.870 | -5.543 | <0.001 |
| **Ethnicity = Asian** | -0.899 | -2.378 | 0.581 | 0.233 | -4.288 | -6.157 | -2.419 | <0.001 | -3.120 | -5.136 | -1.103 | 0.002 |
| **Ethnicity = Black** | 3.307 | 1.082 | 5.531 | 0.004 | 1.684 | -1.136 | 4.504 | 0.242 | 1.951 | -1.603 | 5.507 | 0.282 |
| **Ethnicity = Other** | 0.445 | -0.864 | 1.752 | 0.505 | 0.421 | -1.112 | 1.955 | 0.591 | 1.107 | -0.536 | 2.749 | 0.187 |
| **bs(Time_since_diagnosis, 3)1 * JIA** | -4.742 | -6.971 | -2.518 | <0.001 | -0.918 | -2.925 | 1.087 | 0.370 | 1.900 | 0.395 | 3.406 | 0.013 |
| **bs(Time_since_diagnosis, 3)2 * JIA** | 5.817 | 3.069 | 8.564 | <0.001 | 4.300 | 1.965 | 6.635 | 0.000 | -0.662 | -2.572 | 1.250 | 0.498 |
| **bs(Time_since_diagnosis, 3)3 * JIA** | -0.899 | -4.319 | 2.518 | 0.606 | -1.590 | -4.680 | 1.502 | 0.314 | 0.956 | -1.394 | 3.307 | 0.425 |

Table 10: Linear mixed model results for height measurements in the jSLE cohort

|  | **Whole Cohort** | | | | **Females** | | | | **Males** | | | |
| --- | --- | --- | --- | --- | --- | --- | --- | --- | --- | --- | --- | --- |
|  | Estimate | 95% CI | | p-value | Estimate | 95% CI | | p-value | Estimate | 95% CI | | p-value |
| **Intercept** | 126.163 | 121.612 | 130.855 | <0.001 | 129.886 | 125.431 | 134.462 | <0.001 | 108.810 | 101.184 | 117.158 | <0.001 |
| **bs(Time_since_diagnosis, 3)1** | 21.992 | 17.677 | 26.273 | <0.001 | 17.469 | 12.591 | 22.285 | <0.001 | 34.748 | 26.269 | 42.983 | <0.001 |
| **bs(Time_since_diagnosis, 3)2** | 29.834 | 23.038 | 36.617 | 0.155 | 22.167 | 14.543 | 29.798 | 0.284 | 57.109 | 44.349 | 69.729 | <0.001 |
| **bs(Time_since_diagnosis, 3)3** | 7.780 | -1.401 | 17.002 | 0.090 | -10.021 | -21.392 | 1.453 | 0.072 | 51.139 | 34.598 | 67.437 | <0.001 |
| **jSLE** | -2.249 | -5.729 | 1.256 | 0.209 | -4.645 | -8.163 | -1.123 | 0.010 | 4.926 | -4.354 | 14.478 | 0.315 |
| **Pubertal_Stage = Adolescent** | 34.700 | 30.189 | 39.135 | <0.001 | 27.358 | 22.689 | 31.989 | <0.001 | 51.720 | 41.138 | 61.568 | <0.001 |
| **Pubertal_Stage = Peri-pubertal** | 27.713 | 23.701 | 31.697 | <0.001 | 22.676 | 18.577 | 26.760 | <0.001 | 36.138 | 27.268 | 44.875 | <0.001 |
| **Female** | -4.546 | -7.417 | -1.682 | 0.002 |  |  |  |  |  |  |  |  |
| **Ethnicity = Asian** | -0.298 | -3.655 | 3.055 | 0.863 | -1.975 | -5.465 | 1.519 | 0.271 | 0.283 | -7.729 | 8.205 | 0.944 |
| **Ethnicity = Black** | 2.225 | -2.927 | 7.392 | 0.401 | 0.695 | -4.073 | 5.482 | 0.777 | 7.639 | -8.453 | 23.836 | 0.369 |
| **Ethnicity = Other** | 1.593 | -2.165 | 5.352 | 0.410 | 0.817 | -2.714 | 4.342 | 0.653 | 2.400 | -8.593 | 13.368 | 0.679 |
| **bs(Time_since_diagnosis, 3)1 * jSLE** | -2.830 | -8.819 | 3.161 | 0.355 | 1.490 | -4.700 | 7.703 | 0.635 | -15.974 | -30.828 | -0.823 | 0.040 |
| **bs(Time_since_diagnosis, 3)2 * jSLE** | 7.143 | -4.486 | 18.712 | 0.228 | 6.442 | -4.953 | 17.789 | 0.269 | -6.512 | -36.526 | 22.777 | 0.667 |
| **bs(Time_since_diagnosis, 3)3 * jSLE** | 1.830 | -14.503 | 18.208 | 0.827 | -1.724 | -18.190 | 14.753 | 0.837 | 40.789 | 0.049 | 81.976 | 0.054 |

Table 11: Linear mixed model results for height measurements in the jSLE cohort, stratified by pubertal stage at diagnosis of rheumatic disease.

|  | **Pre-Pubertal** | | | | **Peri-Pubertal** | | | | **Adolescents** | | | |
| --- | --- | --- | --- | --- | --- | --- | --- | --- | --- | --- | --- | --- |
|  | Estimate | 95% CI | | p-value | Estimate | 95% CI | | p-value | Estimate | 95% CI | | p-value |
| **Intercept** | 103.606 | 95.552 | 112.299 | <0.001 | 156.793 | 151.871 | 161.759 | <0.001 | 176.066 | 172.855 | 179.352 | <0.001 |
| **bs(Time_since_diagnosis, 3)1** | 49.704 | 41.878 | 57.632 | <0.001 | 24.001 | 17.295 | 30.832 | <0.001 | 2.254 | -0.800 | 5.282 | 0.148 |
| **bs(Time_since_diagnosis, 3)2** | 70.514 | 62.275 | 79.089 | <0.001 | 18.075 | 12.538 | 23.580 | 0.736 | 2.817 | -0.442 | 6.065 | 0.093 |
| **bs(Time_since_diagnosis, 3)3** | 67.688 | 57.207 | 77.939 | <0.001 | 13.700 | 6.466 | 20.985 | <0.001 | 1.839 | -1.945 | 5.648 | 0.344 |
| **jSLE** | -1.814 | -13.152 | 10.232 | 0.761 | -0.219 | -5.013 | 4.639 | 0.929 | -2.329 | -5.001 | 0.330 | 0.092 |
| **Female** | -1.933 | -8.928 | 4.587 | 0.530 | -9.354 | -12.891 | -5.821 | <0.001 | -13.096 | -16.176 | -10.042 | <0.001 |
| **Ethnicity = Asian** | 2.230 | -6.850 | 10.761 | 0.589 | -4.588 | -8.596 | -0.591 | 0.028 | -4.834 | -8.204 | -1.478 | 0.005 |
| **Ethnicity = Black** | 10.521 | -0.005 | 21.110 | 0.062 | -6.021 | -11.446 | -0.552 | 0.032 | 5.009 | -0.977 | 10.959 | 0.106 |
| **Ethnicity = Other** | 2.677 | -7.484 | 12.844 | 0.620 | -0.406 | -3.946 | 3.128 | 0.824 | 2.376 | -2.060 | 6.824 | 0.302 |
| **bs(Time_since_diagnosis, 3)1 * jSLE** | -7.007 | -18.863 | 4.530 | 0.244 | -8.219 | -16.210 | -0.384 | 0.043 | -1.525 | -5.588 | 2.545 | 0.466 |
| **bs(Time_since_diagnosis, 3)2 * jSLE** | 5.587 | -10.730 | 21.158 | 0.491 | 4.909 | -3.346 | 13.168 | 0.246 | -1.886 | -7.522 | 3.783 | 0.516 |
| **bs(Time_since_diagnosis, 3)3 * jSLE** | -0.074 | -19.631 | 19.162 | 0.994 | -5.709 | -17.350 | 5.840 | 0.337 | 9.789 | 1.638 | 17.806 | 0.018 |

Table 12: Linear mixed model results for weight measurements in the jSLE cohort

|  | **Whole Cohort** | | | | **Females** | | | | **Males** | | | |
| --- | --- | --- | --- | --- | --- | --- | --- | --- | --- | --- | --- | --- |
|  | Estimate | 95% CI | | p-value | Estimate | 95% CI | | p-value | Estimate | 95% CI | | p-value |
| **Intercept** | 23.093 | 18.951 | 27.240 | <0.001 | 21.953 | 17.276 | 26.654 | <0.001 | 16.168 | 10.743 | 21.609 | <0.001 |
| **bs(Time_since_diagnosis, 3)1** | 13.019 | 7.802 | 18.203 | <0.001 | 10.633 | 4.931 | 16.288 | <0.001 | 18.828 | 7.288 | 30.079 | 0.001 |
| **bs(Time_since_diagnosis, 3)2** | 39.627 | 31.283 | 47.973 | <0.001 | 35.937 | 26.570 | 45.281 | <0.001 | 53.209 | 37.005 | 69.847 | <0.001 |
| **bs(Time_since_diagnosis, 3)3** | 38.088 | 26.674 | 49.561 | <0.001 | 29.404 | 16.028 | 42.907 | <0.001 | 56.047 | 36.507 | 75.690 | <0.001 |
| **jSLE** | -2.454 | -5.332 | 0.421 | 0.097 | -1.869 | -5.039 | 1.289 | 0.250 | -6.095 | -12.895 | 0.752 | 0.092 |
| **Pubertal_Stage = Adolescent** | 42.630 | 38.809 | 46.466 | <0.001 | 39.178 | 34.500 | 43.861 | <0.001 | 55.265 | 48.638 | 62.079 | <0.001 |
| **Pubertal_Stage = Peri-pubertal** | 29.589 | 25.733 | 33.449 | <0.001 | 28.037 | 23.361 | 32.707 | <0.001 | 29.125 | 21.987 | 36.236 | <0.001 |
| **Female** | -4.162 | -7.548 | -0.770 | 0.017 |  |  |  |  |  |  |  |  |
| **Ethnicity = Asian** | -2.659 | -6.415 | 1.091 | 0.167 | -6.741 | -11.320 | -2.171 | 0.004 | 5.283 | -0.912 | 11.572 | 0.110 |
| **Ethnicity = Black** | 2.614 | -3.280 | 8.495 | 0.385 | 2.888 | -3.622 | 9.382 | 0.384 | 0.442 | -12.250 | 13.063 | 0.947 |
| **Ethnicity = Other** | -0.753 | -4.642 | 3.126 | 0.705 | -0.027 | -4.248 | 4.177 | 0.990 | -1.133 | -10.668 | 8.630 | 0.822 |
| **bs(Time_since_diagnosis, 3)1 * jSLE** | 12.821 | 5.797 | 19.867 | 0.000 | 11.301 | 4.021 | 18.629 | 0.003 | 17.076 | -1.157 | 34.887 | 0.067 |
| **bs(Time_since_diagnosis, 3)2 * jSLE** | -18.417 | -32.768 | -4.086 | 0.012 | -14.441 | -28.853 | -0.072 | 0.050 | -15.150 | -51.993 | 22.241 | 0.428 |
| **bs(Time_since_diagnosis, 3)3 * jSLE** | 6.538 | -15.054 | 28.281 | 0.554 | 7.457 | -13.476 | 28.468 | 0.487 | 13.922 | -44.771 | 74.412 | 0.649 |

Table 13: Linear mixed model results for weight measurements in the jSLE cohort, stratified by pubertal stage at diagnosis of rheumatic disease.

|  | **Pre-Pubertal** | | | | **Peri-Pubertal** | | | | **Adolescents** | | | |
| --- | --- | --- | --- | --- | --- | --- | --- | --- | --- | --- | --- | --- |
|  | Estimate | 95% CI | | p-value | Estimate | 95% CI | | p-value | Estimate | 95% CI | | p-value |
| **Intercept** | 14.180 | 10.283 | 18.022 | <0.001 | 44.452 | 37.270 | 51.604 | <0.001 | 73.093 | 67.045 | 79.142 | <0.001 |
| **bs(Time_since_diagnosis, 3)1** | 23.207 | 13.617 | 33.417 | <0.001 | 21.106 | 11.822 | 30.447 | <0.001 | 6.654 | 1.323 | 11.937 | 0.014 |
| **bs(Time_since_diagnosis, 3)2** | 55.882 | 44.524 | 66.354 | <0.001 | 24.426 | 17.512 | 31.178 | <0.001 | 3.312 | -2.213 | 8.797 | 0.240 |
| **bs(Time_since_diagnosis, 3)3** | 64.224 | 52.080 | 77.262 | <0.001 | 29.573 | 20.700 | 38.444 | <0.001 | 5.084 | -1.429 | 11.589 | 0.128 |
| **jSLE** | -3.785 | -9.299 | 1.980 | 0.201 | -1.586 | -7.303 | 4.130 | 0.589 | -0.031 | -4.136 | 4.063 | 0.988 |
| **Female** | 3.493 | -0.747 | 7.835 | 0.126 | -0.622 | -6.441 | 5.229 | 0.836 | -12.052 | -17.810 | -6.271 | <0.001 |
| **Ethnicity = Asian** | 8.677 | 3.455 | 13.916 | 0.002 | -3.006 | -8.981 | 2.914 | 0.326 | -6.140 | -12.359 | 0.084 | 0.056 |
| **Ethnicity = Black** | 1.750 | -7.687 | 10.747 | 0.715 | 2.490 | -6.161 | 11.046 | 0.571 | 3.477 | -6.605 | 13.541 | 0.502 |
| **Ethnicity = Other** | 0.929 | -6.601 | 8.389 | 0.814 | 2.745 | -2.692 | 8.167 | 0.328 | -5.160 | -11.704 | 1.350 | 0.122 |
| **bs(Time_since_diagnosis, 3)1 * jSLE** | 0.546 | -15.169 | 14.825 | 0.943 | 4.142 | -6.267 | 14.581 | 0.436 | 4.060 | -2.689 | 10.833 | 0.241 |
| **bs(Time_since_diagnosis, 3)2 * jSLE** | -5.790 | -25.278 | 14.577 | 0.572 | -6.296 | -15.919 | 3.394 | 0.205 | -1.733 | -9.929 | 6.524 | 0.680 |
| **bs(Time_since_diagnosis, 3)3 * jSLE** | 0.909 | -25.047 | 26.473 | 0.946 | -0.843 | -13.342 | 11.808 | 0.896 | 6.535 | -3.907 | 16.997 | 0.223 |

Table 14: Linear mixed model results for height measurements in the JDM cohort

|  | **Whole Cohort** | | | | **Females** | | | | **Males** | | | |
| --- | --- | --- | --- | --- | --- | --- | --- | --- | --- | --- | --- | --- |
|  | Estimate | 95% CI | | p-value | Estimate | 95% CI | | p-value | Estimate | 95% CI | | p-value |
| **Intercept** | 124.223 | 118.761 | 129.812 | <0.001 | 124.805 | 119.791 | 129.944 | <0.001 | 113.578 | 105.275 | 122.466 | <0.001 |
| **bs(Time_since_diagnosis, 3)1** | 28.929 | 23.745 | 34.144 | <0.001 | 27.655 | 21.452 | 33.893 | <0.001 | 35.188 | 26.326 | 43.931 | <0.001 |
| **bs(Time_since_diagnosis, 3)2** | 54.088 | 47.972 | 60.163 | <0.001 | 48.893 | 41.909 | 55.728 | <0.001 | 60.905 | 51.032 | 70.980 | <0.001 |
| **bs(Time_since_diagnosis, 3)3** | 33.661 | 25.338 | 41.921 | <0.001 | 20.888 | 11.255 | 30.461 | <0.001 | 67.793 | 55.201 | 80.091 | <0.001 |
| **JDM** | -1.464 | -6.889 | 4.034 | 0.601 | -2.406 | -8.651 | 4.014 | 0.456 | 3.104 | -6.894 | 13.037 | 0.552 |
| **Pubertal_Stage = Adolescent** | 23.715 | 16.005 | 31.214 | <0.001 | 19.694 | 10.608 | 28.485 | <0.001 | 32.831 | 15.725 | 49.525 | <0.001 |
| **Pubertal_Stage = Peri-pubertal** | 17.801 | 13.636 | 22.019 | <0.001 | 14.986 | 10.132 | 20.034 | <0.001 | 25.498 | 17.407 | 33.124 | <0.001 |
| **Female** | -3.254 | -6.867 | 0.382 | 0.057 |  |  |  |  |  |  |  |  |
| **Ethnicity = Asian** | -8.146 | -13.220 | -3.078 | 0.002 | -4.387 | -10.094 | 1.347 | 0.131 | -14.069 | -23.545 | -4.609 | 0.006 |
| **Ethnicity = Black** | 0.652 | -6.217 | 7.496 | 0.854 | 3.845 | -5.358 | 12.940 | 0.414 | -6.715 | -17.014 | 3.573 | 0.224 |
| **Ethnicity = Other** | 5.081 | -0.985 | 11.153 | 0.106 | 9.695 | 2.598 | 16.796 | 0.009 | -3.551 | -14.515 | 7.243 | 0.534 |
| **bs(Time_since_diagnosis, 3)1 * JDM** | 4.229 | -2.458 | 10.858 | 0.214 | 3.562 | -4.205 | 11.280 | 0.362 | -1.569 | -13.525 | 10.451 | 0.800 |
| **bs(Time_since_diagnosis, 3)2 * JDM** | -8.096 | -17.859 | 1.721 | 0.107 | -9.043 | -20.293 | 2.373 | 0.121 | -4.652 | -19.836 | 10.125 | 0.546 |
| **bs(Time_since_diagnosis, 3)3 * JDM** | 10.266 | -3.248 | 23.642 | 0.136 | 6.162 | -10.007 | 22.107 | 0.453 | 2.403 | -16.657 | 21.196 | 0.807 |

Table 15: Linear mixed model results for height measurements in the JDM cohort, stratified by pubertal stage at diagnosis of rheumatic disease.

|  | **Pre-Pubertal** | | | | **Peri-Pubertal** | | | | **Adolescents** | | | |
| --- | --- | --- | --- | --- | --- | --- | --- | --- | --- | --- | --- | --- |
|  | Estimate | 95% CI | | p-value | Estimate | 95% CI | | p-value | Estimate | 95% CI | | p-value |
| **Intercept** | 116.581 | 109.936 | 123.387 | <0.001 | 148.238 | 142.822 | 153.697 | <0.001 | 172.398 | 163.496 | 185.039 | <0.001 |
| **bs(Time_since_diagnosis, 3)1** | 30.949 | 24.361 | 37.868 | <0.001 | 28.527 | 22.012 | 35.163 | <0.001 | 8.415 | 2.064 | 14.062 | 0.009 |
| **bs(Time_since_diagnosis, 3)2** | 72.160 | 66.169 | 78.147 | <0.001 | 25.329 | 19.008 | 31.677 | <0.001 | -4.922 | -13.098 | 3.328 | 0.254 |
| **bs(Time_since_diagnosis, 3)3** | 52.968 | 45.248 | 60.618 | <0.001 | 22.187 | 14.032 | 30.266 | <0.001 | 3.734 | -4.275 | 11.637 | 0.382 |
| **JDM** | -3.665 | -11.464 | 4.210 | 0.365 | -1.070 | -7.178 | 5.036 | 0.735 | -0.056 | -7.974 | 7.636 | 0.989 |
| **Female** | -3.255 | -8.364 | 1.779 | 0.169 | -6.495 | -10.242 | -2.823 | <0.001 | -10.401 | -21.807 | -2.121 | 0.008 |
| **Ethnicity = Asian** | -8.815 | -15.202 | -2.434 | 0.008 | -1.877 | -8.856 | 5.047 | 0.603 | -4.993 | -11.678 | 1.753 | 0.211 |
| **Ethnicity = Black** | -0.671 | -10.261 | 8.900 | 0.893 | 1.108 | -5.708 | 7.958 | 0.754 | - |  |  |  |
| **Ethnicity = Other** | 2.973 | -8.892 | 14.693 | 0.620 | 5.194 | 0.251 | 10.117 | 0.048 | -4.612 | -13.231 | 2.852 | 0.280 |
| **bs(Time_since_diagnosis, 3)1 * JDM** | 7.517 | -0.863 | 15.623 | 0.074 | -9.765 | -18.081 | -1.560 | 0.021 | -3.185 | -11.594 | 6.192 | 0.488 |
| **bs(Time_since_diagnosis, 3)2 * JDM** | -3.486 | -13.541 | 6.512 | 0.498 | 10.878 | -0.144 | 21.759 | 0.054 | 13.916 | 0.895 | 26.318 | 0.038 |
| **bs(Time_since_diagnosis, 3)3 * JDM** | 12.554 | 0.276 | 24.697 | 0.046 | -12.231 | -26.653 | 2.279 | 0.099 | -12.736 | -29.157 | 4.364 | 0.148 |

Table 16: Linear mixed model results for weight measurements in the JDM cohort

|  | **Whole Cohort** | | | | **Females** | | | | **Males** | | | |
| --- | --- | --- | --- | --- | --- | --- | --- | --- | --- | --- | --- | --- |
|  | Estimate | 95% CI | | p-value | Estimate | 95% CI | | p-value | Estimate | 95% CI | | p-value |
| **Intercept** | 20.873 | 16.249 | 25.480 | <0.001 | 26.403 | 21.501 | 31.314 | <0.001 | 16.832 | 12.119 | 21.482 | <0.001 |
| **bs(Time_since_diagnosis, 3)1** | 15.399 | 9.310 | 21.564 | <0.001 | 17.515 | 10.659 | 24.480 | <0.001 | 14.933 | 4.742 | 25.295 | 0.006 |
| **bs(Time_since_diagnosis, 3)2** | 44.796 | 38.127 | 51.437 | <0.001 | 39.047 | 31.239 | 46.737 | <0.001 | 55.784 | 45.751 | 66.349 | <0.001 |
| **bs(Time_since_diagnosis, 3)3** | 46.401 | 38.292 | 54.517 | <0.001 | 38.702 | 29.128 | 48.257 | <0.001 | 65.920 | 53.958 | 78.351 | <0.001 |
| **JDM** | -2.264 | -6.551 | 2.039 | 0.306 | -3.616 | -9.264 | 2.092 | 0.217 | 2.941 | -2.377 | 8.325 | 0.304 |
| **Pubertal_Stage = Adolescent** | 36.399 | 31.240 | 41.589 | <0.001 | 31.958 | 25.584 | 38.303 | <0.001 | 41.419 | 33.216 | 49.165 | <0.001 |
| **Pubertal_Stage = Peri-pubertal** | 19.433 | 15.924 | 22.981 | <0.001 | 18.014 | 13.725 | 22.341 | <0.001 | 19.514 | 14.745 | 24.145 | <0.001 |
| **Female** | 3.976 | 0.334 | 7.629 | 0.032 |  |  |  |  |  |  |  |  |
| **Ethnicity = Asian** | -1.760 | -6.942 | 3.443 | 0.510 | -1.922 | -8.423 | 4.574 | 0.567 | 0.197 | -6.279 | 6.719 | 0.954 |
| **Ethnicity = Black** | 0.171 | -8.096 | 8.404 | 0.968 | 5.490 | -5.325 | 16.253 | 0.326 | -8.197 | -18.923 | 2.302 | 0.147 |
| **Ethnicity = Other** | 4.127 | -2.155 | 10.401 | 0.204 | 8.622 | 0.358 | 16.803 | 0.043 | -1.220 | -8.348 | 6.235 | 0.750 |
| **bs(Time_since_diagnosis, 3)1 * JDM** | 6.133 | -1.568 | 13.731 | 0.116 | 6.760 | -1.940 | 15.309 | 0.123 | 2.089 | -11.168 | 15.250 | 0.762 |
| **bs(Time_since_diagnosis, 3)2 * JDM** | -1.686 | -11.938 | 8.677 | 0.750 | -3.223 | -15.177 | 8.849 | 0.602 | -2.136 | -19.025 | 14.838 | 0.808 |
| **bs(Time_since_diagnosis, 3)3 * JDM** | 9.113 | -3.981 | 22.133 | 0.173 | 1.307 | -14.397 | 17.003 | 0.871 | 13.028 | -7.244 | 33.250 | 0.215 |

Table 17: Linear mixed model results for weight measurements in the JDM cohort, stratified by pubertal stage at diagnosis of rheumatic disease.

|  | **Pre-Pubertal** | | | | **Peri-Pubertal** | | | | **Adolescents** | | | |
| --- | --- | --- | --- | --- | --- | --- | --- | --- | --- | --- | --- | --- |
|  | Estimate | 95% CI | | p-value | Estimate | 95% CI | | p-value | Estimate | 95% CI | | p-value |
| **Intercept** | 16.378 | 11.922 | 20.720 | <0.001 | 37.855 | 29.607 | 46.146 | <0.001 | 78.366 | 61.065 | 95.180 | <0.001 |
| **bs(Time_since_diagnosis, 3)1** | 18.465 | 10.679 | 26.652 | <0.001 | 27.853 | 18.749 | 37.475 | <0.001 | -10.199 | -22.527 | 1.870 | 0.113 |
| **bs(Time_since_diagnosis, 3)2** | 55.030 | 48.060 | 62.059 | <0.001 | 17.630 | 9.408 | 25.699 | <0.001 | 2.790 | -10.023 | 15.510 | 0.682 |
| **bs(Time_since_diagnosis, 3)3** | 55.236 | 46.922 | 63.825 | <0.001 | 37.018 | 27.520 | 46.403 | <0.001 | 0.606 | -13.299 | 14.752 | 0.935 |
| **JDM** | 1.685 | -3.032 | 6.484 | 0.498 | 0.278 | -7.637 | 8.302 | 0.946 | -15.140 | -26.271 | -3.646 | 0.019 |
| **Female** | 5.087 | 1.471 | 8.717 | 0.008 | 3.002 | -3.463 | 9.344 | 0.355 | -7.780 | -22.616 | 7.453 | 0.341 |
| **Ethnicity = Asian** | -0.751 | -5.924 | 4.463 | 0.782 | -5.413 | -16.063 | 5.108 | 0.318 | 3.024 | -11.949 | 17.919 | 0.708 |
| **Ethnicity = Black** | 0.012 | -8.543 | 8.524 | 0.998 | 2.067 | -10.805 | 14.900 | 0.757 |  |  |  |  |
| **Ethnicity = Other** | 4.169 | -3.063 | 11.403 | 0.274 | 2.285 | -7.136 | 11.642 | 0.640 | -9.341 | -32.448 | 12.991 | 0.409 |
| **bs(Time_since_diagnosis, 3)1 * JDM** | -1.186 | -11.491 | 8.761 | 0.819 | -7.599 | -18.689 | 2.990 | 0.163 | 24.072 | 7.151 | 40.562 | 0.006 |
| **bs(Time_since_diagnosis, 3)2 * JDM** | -2.938 | -13.915 | 8.211 | 0.607 | 1.085 | -9.955 | 12.255 | 0.849 | -4.292 | -25.665 | 17.719 | 0.706 |
| **bs(Time_since_diagnosis, 3)3 * JDM** | 0.836 | -12.774 | 14.094 | 0.904 | 1.089 | -11.960 | 14.219 | 0.872 | 51.006 | 20.759 | 79.695 | 0.001 |

**Results of analyses using standardised height and weight z-scores**

Table 18: Linear mixed model results for height Z-scores in the JIA cohort

|  | **Whole Cohort** | | | | **Females** | | | | **Males** | | | |
| --- | --- | --- | --- | --- | --- | --- | --- | --- | --- | --- | --- | --- |
|  | Estimate | 95% CI | | p-value | Estimate | 95% CI | | p-value | Estimate | 95% CI | | p-value |
| **Intercept** | 0.258 | 0.196 | 0.320 | <0.001 | 0.248 | 0.181 | 0.316 | <0.001 | 0.258 | 0.162 | 0.353 | <0.001 |
| **bs(Time_since_diagnosis, 3)1** | 0.082 | -0.009 | 0.172 | 0.076 | 0.106 | 0.000 | 0.212 | 0.050 | 0.005 | -0.167 | 0.177 | 0.955 |
| **bs(Time_since_diagnosis, 3)2** | -0.129 | -0.263 | 0.005 | 0.059 | -0.184 | -0.336 | -0.031 | 0.018 | 0.065 | -0.211 | 0.341 | 0.646 |
| **bs(Time_since_diagnosis, 3)3** | -0.493 | -0.666 | -0.320 | <0.001 | -0.409 | -0.597 | -0.221 | <0.001 | -0.804 | -1.196 | -0.412 | <0.001 |
| **JIA** | -0.061 | -0.121 | -0.002 | 0.043 | -0.031 | -0.101 | 0.039 | 0.387 | -0.122 | -0.233 | -0.011 | 0.032 |
| **Pubertal_Stage = Adolescent** | -0.042 | -0.100 | 0.017 | 0.166 | -0.034 | -0.103 | 0.035 | 0.336 | -0.087 | -0.205 | 0.030 | 0.145 |
| **Pubertal_Stage = Peri-pubertal** | -0.044 | -0.097 | 0.009 | 0.100 | -0.055 | -0.121 | 0.010 | 0.096 | -0.021 | -0.113 | 0.071 | 0.658 |
| **Female** | -0.011 | -0.059 | 0.038 | 0.668 |  |  |  |  |  |  |  |  |
| **Ethnicity = Asian** | -0.380 | -0.477 | -0.284 | <0.001 | -0.488 | -0.610 | -0.366 | <0.001 | -0.212 | -0.370 | -0.055 | 0.008 |
| **Ethnicity = Black** | 0.167 | 0.015 | 0.319 | 0.031 | 0.083 | -0.110 | 0.275 | 0.402 | 0.302 | 0.055 | 0.549 | 0.017 |
| **Ethnicity = Other** | 0.025 | -0.056 | 0.105 | 0.550 | 0.021 | -0.075 | 0.117 | 0.665 | 0.030 | -0.119 | 0.179 | 0.691 |
| **bs(Time_since_diagnosis, 3)1 * JIA** | -0.235 | -0.363 | -0.107 | <0.001 | -0.297 | -0.448 | -0.147 | <0.001 | -0.098 | -0.338 | 0.142 | 0.424 |
| **bs(Time_since_diagnosis, 3)2 * JIA** | 0.138 | -0.081 | 0.358 | 0.217 | 0.110 | -0.144 | 0.363 | 0.396 | 0.146 | -0.283 | 0.574 | 0.506 |
| **bs(Time_since_diagnosis, 3)3 * JIA** | 0.168 | -0.139 | 0.476 | 0.284 | 0.470 | 0.122 | 0.817 | 0.008 | -0.423 | -1.051 | 0.207 | 0.188 |

Table 19: Linear mixed model results for height Z-scores in the JIA cohort, stratified by pubertal stage at diagnosis of rheumatic disease.

|  | **Pre-Pubertal** | | | | **Peri-Pubertal** | | | | **Adolescents** | | | |
| --- | --- | --- | --- | --- | --- | --- | --- | --- | --- | --- | --- | --- |
|  | Estimate | 95% CI | | p-value | Estimate | 95% CI | | p-value | Estimate | 95% CI | | p-value |
| **Intercept** | 0.096 | -0.008 | 0.200 | 0.072 | 0.369 | 0.284 | 0.455 | 0.000 | 0.161 | 0.057 | 0.265 | 0.002 |
| **bs(Time_since_diagnosis, 3)1** | 0.571 | 0.392 | 0.750 | <0.001 | -0.158 | -0.287 | -0.030 | 0.016 | 0.014 | -0.099 | 0.128 | 0.804 |
| **bs(Time_since_diagnosis, 3)2** | -0.204 | -0.376 | -0.031 | 0.020 | -0.408 | -0.540 | -0.276 | <0.001 | 0.122 | -0.004 | 0.247 | 0.057 |
| **bs(Time_since_diagnosis, 3)3** | -0.105 | -0.316 | 0.106 | 0.329 | -0.018 | -0.189 | 0.153 | 0.838 | 0.114 | -0.032 | 0.260 | 0.127 |
| **JIA** | -0.197 | -0.325 | -0.068 | 0.003 | -0.061 | -0.158 | 0.035 | 0.214 | 0.017 | -0.076 | 0.110 | 0.723 |
| **Female** | -0.007 | -0.086 | 0.072 | 0.858 | -0.022 | -0.095 | 0.052 | 0.558 | 0.012 | -0.090 | 0.113 | 0.821 |
| **Ethnicity = Asian** | -0.184 | -0.339 | -0.030 | 0.019 | -0.426 | -0.578 | -0.273 | <0.001 | -0.668 | -0.861 | -0.475 | <0.001 |
| **Ethnicity = Black** | 0.358 | 0.115 | 0.602 | 0.004 | 0.135 | -0.095 | 0.365 | 0.250 | -0.241 | -0.573 | 0.091 | 0.155 |
| **Ethnicity = Other** | 0.137 | 0.007 | 0.267 | 0.039 | 0.014 | -0.112 | 0.140 | 0.828 | -0.045 | -0.205 | 0.116 | 0.585 |
| **bs(Time_since_diagnosis, 3)1 * JIA** | -0.388 | -0.625 | -0.150 | 0.001 | 0.032 | -0.144 | 0.209 | 0.719 | -0.053 | -0.216 | 0.109 | 0.520 |
| **bs(Time_since_diagnosis, 3)2 * JIA** | 0.540 | 0.256 | 0.823 | <0.001 | -0.042 | -0.262 | 0.177 | 0.707 | -0.124 | -0.324 | 0.077 | 0.226 |
| **bs(Time_since_diagnosis, 3)3 * JIA** | 0.112 | -0.246 | 0.470 | 0.538 | -0.172 | -0.467 | 0.124 | 0.255 | -0.165 | -0.400 | 0.069 | 0.168 |

Table 20: Linear mixed model results for weight Z-scores in the JIA cohort

|  | **Whole Cohort** | | | | **Females** | | | | **Males** | | | |
| --- | --- | --- | --- | --- | --- | --- | --- | --- | --- | --- | --- | --- |
|  | Estimate | 95% CI | | p-value | Estimate | 95% CI | | p-value | Estimate | 95% CI | | p-value |
| **Intercept** | 0.134 | 0.067 | 0.201 | <0.001 | 0.019 | -0.051 | 0.089 | 0.593 | 0.288 | 0.185 | 0.391 | <0.001 |
| **bs(Time_since_diagnosis, 3)1** | 0.350 | 0.257 | 0.443 | <0.001 | 0.426 | 0.318 | 0.533 | <0.001 | 0.042 | -0.145 | 0.228 | 0.662 |
| **bs(Time_since_diagnosis, 3)2** | 0.335 | 0.193 | 0.476 | <0.001 | 0.377 | 0.216 | 0.539 | <0.001 | 0.492 | 0.195 | 0.787 | 0.001 |
| **bs(Time_since_diagnosis, 3)3** | 0.541 | 0.362 | 0.721 | <0.001 | 0.894 | 0.696 | 1.091 | <0.001 | -0.915 | -1.325 | -0.504 | <0.001 |
| **JIA** | 0.035 | -0.023 | 0.093 | 0.238 | 0.066 | -0.002 | 0.133 | 0.057 | -0.051 | -0.167 | 0.064 | 0.382 |
| **Pubertal_Stage = Adolescent** | 0.292 | 0.232 | 0.352 | <0.001 | 0.363 | 0.293 | 0.432 | <0.001 | 0.035 | -0.091 | 0.162 | 0.581 |
| **Pubertal_Stage = Peri-pubertal** | 0.247 | 0.189 | 0.306 | <0.001 | 0.252 | 0.181 | 0.323 | <0.001 | 0.263 | 0.159 | 0.368 | <0.001 |
| **Female** | -0.046 | -0.100 | 0.007 | 0.090 |  |  |  |  |  |  |  |  |
| **Ethnicity = Asian** | -0.323 | -0.424 | -0.223 | <0.001 | -0.342 | -0.467 | -0.218 | <0.001 | -0.276 | -0.445 | -0.107 | 0.001 |
| **Ethnicity = Black** | 0.332 | 0.175 | 0.490 | <0.001 | 0.397 | 0.200 | 0.594 | <0.001 | 0.226 | -0.036 | 0.488 | 0.091 |
| **Ethnicity = Other** | 0.095 | 0.011 | 0.180 | 0.027 | 0.117 | 0.018 | 0.216 | 0.021 | 0.038 | -0.122 | 0.198 | 0.641 |
| **bs(Time_since_diagnosis, 3)1 * JIA** | -0.044 | -0.174 | 0.087 | 0.512 | -0.117 | -0.269 | 0.035 | 0.130 | 0.227 | -0.025 | 0.480 | 0.077 |
| **bs(Time_since_diagnosis, 3)2 * JIA** | 0.163 | -0.067 | 0.393 | 0.165 | 0.255 | -0.011 | 0.521 | 0.060 | -0.252 | -0.704 | 0.200 | 0.273 |
| **bs(Time_since_diagnosis, 3)3 * JIA** | -0.253 | -0.567 | 0.061 | 0.115 | 0.118 | -0.237 | 0.473 | 0.515 | -1.085 | -1.741 | -0.427 | 0.001 |

Table 21: Linear mixed model results for weight Z-scores in the JIA cohort, stratified by pubertal stage at diagnosis of rheumatic disease.

|  | **Pre-Pubertal** | | | | **Peri-Pubertal** | | | | **Adolescents** | | | |
| --- | --- | --- | --- | --- | --- | --- | --- | --- | --- | --- | --- | --- |
|  | Estimate | 95% CI | | p-value | Estimate | 95% CI | | p-value | Estimate | 95% CI | | p-value |
| **Intercept** | 0.152 | 0.048 | 0.256 | 0.004 | 0.558 | 0.461 | 0.655 | <0.001 | 0.230 | 0.115 | 0.345 | <0.001 |
| **bs(Time_since_diagnosis, 3)1** | 0.578 | 0.398 | 0.757 | <0.001 | 0.031 | -0.113 | 0.175 | 0.672 | 0.069 | -0.038 | 0.177 | 0.207 |
| **bs(Time_since_diagnosis, 3)2** | 0.167 | -0.008 | 0.342 | 0.061 | -0.116 | -0.253 | 0.021 | 0.097 | 0.199 | 0.076 | 0.322 | 0.002 |
| **bs(Time_since_diagnosis, 3)3** | 0.615 | 0.408 | 0.822 | <0.001 | 0.484 | 0.307 | 0.662 | <0.001 | 0.648 | 0.501 | 0.794 | <0.001 |
| **JIA** | -0.199 | -0.322 | -0.075 | 0.002 | 0.013 | -0.091 | 0.117 | 0.810 | 0.110 | 0.018 | 0.201 | 0.019 |
| **Female** | -0.066 | -0.152 | 0.019 | 0.128 | -0.102 | -0.184 | -0.019 | 0.016 | 0.123 | 0.012 | 0.234 | 0.030 |
| **Ethnicity = Asian** | -0.274 | -0.432 | -0.117 | 0.001 | -0.370 | -0.534 | -0.205 | <0.001 | -0.368 | -0.561 | -0.176 | <0.001 |
| **Ethnicity = Black** | 0.410 | 0.172 | 0.647 | 0.001 | 0.309 | 0.061 | 0.557 | 0.015 | 0.153 | -0.186 | 0.492 | 0.377 |
| **Ethnicity = Other** | 0.119 | -0.019 | 0.257 | 0.092 | 0.089 | -0.046 | 0.224 | 0.197 | 0.093 | -0.064 | 0.250 | 0.248 |
| **bs(Time_since_diagnosis, 3)1 * JIA** | 0.010 | -0.227 | 0.248 | 0.932 | 0.157 | -0.032 | 0.346 | 0.104 | 0.118 | -0.037 | 0.273 | 0.136 |
| **bs(Time_since_diagnosis, 3)2 * JIA** | 0.546 | 0.260 | 0.831 | <0.001 | -0.065 | -0.279 | 0.149 | 0.553 | -0.168 | -0.362 | 0.027 | 0.091 |
| **bs(Time_since_diagnosis, 3)3 * JIA** | -0.051 | -0.401 | 0.300 | 0.777 | -0.004 | -0.285 | 0.276 | 0.976 | 0.084 | -0.153 | 0.322 | 0.486 |

Table 22: Linear mixed model results for height Z-scores in the jSLE cohort

|  | **Whole Cohort** | | | | **Females** | | | | **Males** | | | |
| --- | --- | --- | --- | --- | --- | --- | --- | --- | --- | --- | --- | --- |
|  | Estimate | 95% CI | | p-value | Estimate | 95% CI | | p-value | Estimate | 95% CI | | p-value |
| **Intercept** | 0.354 | -0.004 | 0.714 | 0.055 | 0.334 | -0.054 | 0.722 | 0.095 | 0.606 | 0.049 | 1.172 | 0.042 |
| **bs(Time_since_diagnosis, 3)1** | 0.232 | -0.242 | 0.708 | 0.340 | 0.495 | -0.046 | 1.038 | 0.075 | -0.392 | -1.339 | 0.553 | 0.422 |
| **bs(Time_since_diagnosis, 3)2** | -0.640 | -1.298 | 0.018 | 0.057 | -0.590 | -1.404 | 0.225 | 0.157 | -0.500 | -1.575 | 0.625 | 0.373 |
| **bs(Time_since_diagnosis, 3)3** | -0.152 | -0.995 | 0.692 | 0.725 | -0.027 | -1.150 | 1.092 | 0.963 | -0.531 | -1.717 | 0.673 | 0.396 |
| **jSLE** | -0.247 | -0.534 | 0.041 | 0.095 | -0.253 | -0.570 | 0.065 | 0.121 | 0.034 | -0.660 | 0.756 | 0.926 |
| **Pubertal_Stage = Adolescent** | -0.202 | -0.517 | 0.112 | 0.213 | -0.243 | -0.613 | 0.127 | 0.203 | -0.198 | -0.833 | 0.430 | 0.553 |
| **Pubertal_Stage = Peri-pubertal** | -0.120 | -0.432 | 0.191 | 0.450 | -0.189 | -0.554 | 0.177 | 0.311 | -0.038 | -0.635 | 0.557 | 0.905 |
| **Female** | 0.023 | -0.258 | 0.303 | 0.875 |  |  |  |  |  |  |  |  |
| **Ethnicity = Asian** | -0.469 | -0.800 | -0.141 | 0.005 | -0.400 | -0.793 | -0.007 | 0.048 | -0.812 | -1.446 | -0.201 | 0.011 |
| **Ethnicity = Black** | 0.247 | -0.246 | 0.740 | 0.331 | 0.036 | -0.495 | 0.567 | 0.896 | 1.055 | -0.195 | 2.299 | 0.112 |
| **Ethnicity = Other** | 0.210 | -0.158 | 0.578 | 0.269 | 0.288 | -0.115 | 0.692 | 0.166 | -0.282 | -1.133 | 0.547 | 0.526 |
| **bs(Time_since_diagnosis, 3)1 * jSLE** | -0.995 | -1.652 | -0.340 | 0.003 | -1.043 | -1.741 | -0.346 | 0.004 | -1.156 | -2.720 | 0.448 | 0.159 |
| **bs(Time_since_diagnosis, 3)2 * jSLE** | 1.326 | 0.115 | 2.530 | 0.031 | 1.697 | 0.427 | 2.960 | 0.009 | -0.831 | -3.656 | 1.849 | 0.551 |
| **bs(Time_since_diagnosis, 3)3 * jSLE** | 0.163 | -1.517 | 1.836 | 0.849 | -0.479 | -2.236 | 1.279 | 0.594 | 1.880 | -2.052 | 5.672 | 0.348 |

Table 23: Linear mixed model results for height Z-scores in the jSLE cohort, stratified by pubertal stage at diagnosis of rheumatic disease.

|  | **Pre-Pubertal** | | | | **Peri-Pubertal** | | | | **Adolescents** | | | |
| --- | --- | --- | --- | --- | --- | --- | --- | --- | --- | --- | --- | --- |
|  | Estimate | 95% CI | | p-value | Estimate | 95% CI | | p-value | Estimate | 95% CI | | p-value |
| **Intercept** | 0.243 | -0.353 | 0.853 | 0.438 | 0.477 | -0.126 | 1.071 | 0.121 | 0.213 | -0.271 | 0.697 | 0.395 |
| **bs(Time_since_diagnosis, 3)1** | 0.082 | -0.951 | 1.134 | 0.878 | 0.052 | -0.827 | 0.954 | 0.907 | 0.147 | -0.314 | 0.607 | 0.533 |
| **bs(Time_since_diagnosis, 3)2** | -0.539 | -1.423 | 0.353 | 0.241 | -0.460 | -1.107 | 0.185 | 0.165 | 0.128 | -0.364 | 0.619 | 0.613 |
| **bs(Time_since_diagnosis, 3)3** | -0.321 | -1.331 | 0.701 | 0.542 | -0.312 | -1.125 | 0.505 | 0.454 | 0.303 | -0.267 | 0.873 | 0.301 |
| **jSLE** | -0.608 | -1.396 | 0.206 | 0.145 | -0.021 | -0.548 | 0.513 | 0.938 | -0.303 | -0.700 | 0.093 | 0.141 |
| **Female** | 0.178 | -0.331 | 0.683 | 0.505 | -0.035 | -0.494 | 0.426 | 0.884 | -0.151 | -0.609 | 0.309 | 0.526 |
| **Ethnicity = Asian** | -0.174 | -0.887 | 0.511 | 0.621 | -0.526 | -1.064 | 0.011 | 0.058 | -0.664 | -1.185 | -0.145 | 0.013 |
| **Ethnicity = Black** | 1.217 | 0.314 | 2.113 | 0.012 | -0.599 | -1.305 | 0.106 | 0.102 | 0.727 | -0.183 | 1.635 | 0.123 |
| **Ethnicity = Other** | 0.777 | -0.184 | 1.735 | 0.121 | -0.203 | -0.675 | 0.269 | 0.406 | 0.399 | -0.286 | 1.086 | 0.261 |
| **bs(Time_since_diagnosis, 3)1 * jSLE** | -0.503 | -2.040 | 1.003 | 0.519 | -1.010 | -2.065 | 0.019 | 0.055 | -0.453 | -1.073 | 0.168 | 0.155 |
| **bs(Time_since_diagnosis, 3)2 * jSLE** | 0.793 | -0.978 | 2.511 | 0.371 | 0.027 | -0.991 | 1.050 | 0.958 | -0.289 | -1.144 | 0.567 | 0.511 |
| **bs(Time_since_diagnosis, 3)3 * jSLE** | 0.449 | -1.628 | 2.523 | 0.676 | -0.123 | -1.540 | 1.295 | 0.866 | 1.383 | 0.151 | 2.601 | 0.028 |

Table 24: Linear mixed model results for weight Z-scores in the jSLE cohort

|  | **Whole Cohort** | | | | **Females** | | | | **Males** | | | |
| --- | --- | --- | --- | --- | --- | --- | --- | --- | --- | --- | --- | --- |
|  | Estimate | 95% CI | | p-value | Estimate | 95% CI | | p-value | Estimate | 95% CI | | p-value |
| **Intercept** | 0.488 | 0.107 | 0.870 | 0.013 | 0.202 | -0.229 | 0.635 | 0.362 | 0.590 | 0.056 | 1.124 | 0.036 |
| **bs(Time_since_diagnosis, 3)1** | -0.126 | -0.625 | 0.372 | 0.621 | 0.142 | -0.403 | 0.685 | 0.609 | -0.654 | -1.770 | 0.468 | 0.256 |
| **bs(Time_since_diagnosis, 3)2** | 1.178 | 0.392 | 1.961 | 0.003 | 0.937 | 0.059 | 1.810 | 0.037 | 1.195 | -0.479 | 2.870 | 0.167 |
| **bs(Time_since_diagnosis, 3)3** | -0.590 | -1.672 | 0.485 | 0.284 | 0.088 | -1.165 | 1.337 | 0.891 | -1.515 | -3.634 | 0.592 | 0.166 |
| **jSLE** | -0.089 | -0.368 | 0.189 | 0.531 | 0.054 | -0.251 | 0.360 | 0.729 | -0.487 | -1.175 | 0.208 | 0.181 |
| **Pubertal_Stage = Adolescent** | 0.264 | -0.078 | 0.605 | 0.131 | 0.226 | -0.198 | 0.648 | 0.299 | 0.432 | -0.157 | 1.019 | 0.164 |
| **Pubertal_Stage = Peri-pubertal** | 0.252 | -0.100 | 0.605 | 0.161 | 0.231 | -0.202 | 0.663 | 0.298 | 0.215 | -0.411 | 0.845 | 0.509 |
| **Female** | -0.233 | -0.550 | 0.085 | 0.152 |  |  |  |  |  |  |  |  |
| **Ethnicity = Asian** | -0.497 | -0.849 | -0.146 | 0.006 | -0.768 | -1.204 | -0.332 | 0.001 | -0.048 | -0.601 | 0.510 | 0.870 |
| **Ethnicity = Black** | 0.483 | -0.067 | 1.032 | 0.087 | 0.518 | -0.102 | 1.139 | 0.103 | 0.304 | -0.836 | 1.449 | 0.614 |
| **Ethnicity = Other** | -0.008 | -0.372 | 0.355 | 0.965 | 0.083 | -0.321 | 0.485 | 0.686 | -0.385 | -1.236 | 0.477 | 0.394 |
| **bs(Time_since_diagnosis, 3)1 * jSLE** | 0.737 | 0.062 | 1.412 | 0.033 | 0.349 | -0.351 | 1.052 | 0.331 | 1.670 | -0.162 | 3.465 | 0.073 |
| **bs(Time_since_diagnosis, 3)2 * jSLE** | -2.371 | -3.728 | -1.017 | 0.001 | -1.783 | -3.143 | -0.430 | 0.010 | -2.750 | -6.514 | 1.091 | 0.152 |
| **bs(Time_since_diagnosis, 3)3 * jSLE** | 1.587 | -0.490 | 3.677 | 0.136 | 1.257 | -0.750 | 3.273 | 0.222 | -0.493 | -6.332 | 5.354 | 0.870 |

Table 25: Linear mixed model results for weight Z-scores in the jSLE cohort, stratified by pubertal stage at diagnosis of rheumatic disease.

|  | **Pre-Pubertal** | | | | **Peri-Pubertal** | | | | **Adolescents** | | | |
| --- | --- | --- | --- | --- | --- | --- | --- | --- | --- | --- | --- | --- |
|  | Estimate | 95% CI | | p-value | Estimate | 95% CI | | p-value | Estimate | 95% CI | | p-value |
| **Intercept** | 0.520 | -0.015 | 1.063 | 0.066 | 0.809 | 0.121 | 1.496 | 0.022 | 0.789 | 0.217 | 1.361 | 0.008 |
| **bs(Time_since_diagnosis, 3)1** | -0.180 | -1.270 | 0.902 | 0.744 | -0.637 | -1.545 | 0.275 | 0.167 | 0.414 | -0.101 | 0.927 | 0.116 |
| **bs(Time_since_diagnosis, 3)2** | 1.194 | 0.023 | 2.359 | 0.047 | 0.120 | -0.530 | 0.764 | 0.718 | -0.037 | -0.574 | 0.496 | 0.892 |
| **bs(Time_since_diagnosis, 3)3** | -0.720 | -2.058 | 0.618 | 0.299 | 0.217 | -0.627 | 1.048 | 0.612 | 0.246 | -0.391 | 0.882 | 0.452 |
| **jSLE** | -0.924 | -1.671 | -0.169 | 0.020 | -0.261 | -0.803 | 0.284 | 0.348 | 0.132 | -0.266 | 0.529 | 0.520 |
| **Female** | -0.007 | -0.515 | 0.502 | 0.978 | -0.058 | -0.614 | 0.495 | 0.840 | -0.448 | -0.986 | 0.092 | 0.107 |
| **Ethnicity = Asian** | -0.119 | -0.775 | 0.540 | 0.728 | -0.413 | -0.979 | 0.150 | 0.157 | -0.745 | -1.327 | -0.162 | 0.013 |
| **Ethnicity = Black** | 0.607 | -0.416 | 1.635 | 0.260 | 0.500 | -0.320 | 1.313 | 0.233 | 0.412 | -0.533 | 1.356 | 0.397 |
| **Ethnicity = Other** | 0.303 | -0.529 | 1.133 | 0.487 | 0.125 | -0.395 | 0.641 | 0.641 | -0.419 | -1.035 | 0.194 | 0.180 |
| **bs(Time_since_diagnosis, 3)1 * jSLE** | 0.821 | -0.807 | 2.433 | 0.324 | 1.374 | 0.348 | 2.398 | 0.008 | 0.198 | -0.455 | 0.851 | 0.554 |
| **bs(Time_since_diagnosis, 3)2 * jSLE** | -1.004 | -3.164 | 1.141 | 0.366 | -1.508 | -2.427 | -0.588 | 0.001 | -0.290 | -1.084 | 0.508 | 0.477 |
| **bs(Time_since_diagnosis, 3)3 * jSLE** | 1.185 | -1.629 | 3.958 | 0.411 | 0.377 | -0.797 | 1.570 | 0.534 | 0.507 | -0.510 | 1.524 | 0.331 |

Table 26: Linear mixed model results for height Z-scores in the JDM cohort

|  | **Whole Cohort** | | | | **Females** | | | | **Males** | | | |
| --- | --- | --- | --- | --- | --- | --- | --- | --- | --- | --- | --- | --- |
|  | Estimate | 95% CI | | p-value | Estimate | 95% CI | | p-value | Estimate | 95% CI | | p-value |
| **Intercept** | 0.565 | 0.151 | 0.977 | 0.008 | 0.299 | -0.091 | 0.686 | 0.138 | 0.903 | 0.232 | 1.593 | 0.011 |
| **bs(Time_since_diagnosis, 3)1** | -0.135 | -0.639 | 0.373 | 0.601 | -0.112 | -0.711 | 0.495 | 0.717 | -0.185 | -1.090 | 0.712 | 0.690 |
| **bs(Time_since_diagnosis, 3)2** | -0.365 | -0.882 | 0.154 | 0.169 | -0.093 | -0.676 | 0.490 | 0.756 | -0.885 | -1.810 | 0.076 | 0.070 |
| **bs(Time_since_diagnosis, 3)3** | -0.451 | -1.075 | 0.178 | 0.161 | -0.315 | -1.012 | 0.392 | 0.384 | -0.696 | -1.796 | 0.405 | 0.226 |
| **JDM** | -0.287 | -0.684 | 0.110 | 0.162 | -0.240 | -0.690 | 0.209 | 0.304 | -0.403 | -1.192 | 0.381 | 0.332 |
| **Pubertal_Stage = Adolescent** | -0.253 | -0.793 | 0.287 | 0.366 | -0.029 | -0.593 | 0.537 | 0.922 | -0.758 | -2.114 | 0.610 | 0.295 |
| **Pubertal_Stage = Peri-pubertal** | -0.174 | -0.481 | 0.134 | 0.273 | -0.100 | -0.458 | 0.259 | 0.590 | -0.312 | -0.877 | 0.249 | 0.287 |
| **Female** | -0.096 | -0.415 | 0.223 | 0.560 |  |  |  |  |  |  |  |  |
| **Ethnicity = Asian** | -0.422 | -0.905 | 0.061 | 0.089 | -0.377 | -0.938 | 0.187 | 0.196 | -0.531 | -1.389 | 0.328 | 0.236 |
| **Ethnicity = Black** | 0.208 | -0.472 | 0.889 | 0.555 | 0.343 | -0.626 | 1.306 | 0.493 | -0.032 | -0.962 | 0.901 | 0.948 |
| **Ethnicity = Other** | 0.374 | -0.204 | 0.953 | 0.213 | 1.365 | 0.638 | 2.095 | <0.001 | -1.022 | -1.947 | -0.108 | 0.038 |
| **bs(Time_since_diagnosis, 3)1 * JDM** | 0.142 | -0.503 | 0.785 | 0.666 | -0.028 | -0.778 | 0.718 | 0.941 | 0.570 | -0.642 | 1.787 | 0.363 |
| **bs(Time_since_diagnosis, 3)2 * JDM** | 0.047 | -0.792 | 0.888 | 0.914 | -0.095 | -1.062 | 0.881 | 0.850 | 0.395 | -1.059 | 1.833 | 0.596 |
| **bs(Time_since_diagnosis, 3)3 * JDM** | -0.269 | -1.318 | 0.773 | 0.617 | -0.370 | -1.573 | 0.819 | 0.548 | 0.084 | -1.695 | 1.851 | 0.928 |

Table 27: Linear mixed model results for height Z-scores in the JDM cohort, stratified by pubertal stage at diagnosis of rheumatic disease.

|  | **Pre-Pubertal** | | | | **Peri-Pubertal** | | | | **Adolescents** | | | |
| --- | --- | --- | --- | --- | --- | --- | --- | --- | --- | --- | --- | --- |
|  | Estimate | 95% CI | | p-value | Estimate | 95% CI | | p-value | Estimate | 95% CI | | p-value |
| **Intercept** | 0.650 | 0.061 | 1.237 | 0.034 | 0.204 | -0.375 | 0.783 | 0.500 | -0.414 | -1.705 | 1.592 | 0.529 |
| **bs(Time_since_diagnosis, 3)1** | -0.204 | -0.974 | 0.583 | 0.609 | 0.517 | -0.233 | 1.272 | 0.182 | 0.998 | 0.276 | 1.634 | 0.006 |
| **bs(Time_since_diagnosis, 3)2** | -0.171 | -0.778 | 0.448 | 0.587 | -0.693 | -1.343 | -0.033 | 0.041 | -0.686 | -1.375 | 0.029 | 0.079 |
| **bs(Time_since_diagnosis, 3)3** | -0.419 | -1.159 | 0.334 | 0.277 | -0.026 | -0.787 | 0.737 | 0.948 | 0.175 | -0.479 | 0.783 | 0.601 |
| **JDM** | -0.227 | -0.876 | 0.428 | 0.501 | -0.242 | -0.806 | 0.320 | 0.411 | 0.248 | -0.730 | 1.170 | 0.618 |
| **Female** | -0.259 | -0.695 | 0.180 | 0.258 | 0.049 | -0.439 | 0.536 | 0.847 | 0.365 | -1.533 | 1.637 | 0.532 |
| **Ethnicity = Asian** | -0.689 | -1.294 | -0.081 | 0.029 | 0.189 | -0.759 | 1.135 | 0.698 | -0.833 | -1.952 | 0.280 | 0.211 |
| **Ethnicity = Black** | -0.118 | -1.048 | 0.809 | 0.807 | 0.954 | -0.042 | 1.938 | 0.068 |  |  |  |  |
| **Ethnicity = Other** | 0.220 | -0.862 | 1.302 | 0.693 | 0.671 | -0.067 | 1.410 | 0.086 | -0.380 | -1.989 | 0.894 | 0.592 |
| **bs(Time_since_diagnosis, 3)1 * JDM** | 0.484 | -0.469 | 1.425 | 0.320 | -1.308 | -2.273 | -0.362 | 0.008 | -0.764 | -1.775 | 0.373 | 0.169 |
| **bs(Time_since_diagnosis, 3)2 * JDM** | -0.126 | -1.143 | 0.882 | 0.809 | 1.610 | 0.415 | 2.825 | 0.008 | 1.012 | -0.338 | 2.282 | 0.149 |
| **bs(Time_since_diagnosis, 3)3 * JDM** | 0.035 | -1.158 | 1.209 | 0.954 | -2.132 | -3.588 | -0.684 | 0.005 | -0.739 | -2.529 | 1.181 | 0.433 |

Table 28: Linear mixed model results for weight Z-scores in the JDM cohort

|  | **Whole Cohort** | | | | **Females** | | | | **Males** | | | |
| --- | --- | --- | --- | --- | --- | --- | --- | --- | --- | --- | --- | --- |
|  | Estimate | 95% CI | | p-value | Estimate | 95% CI | | p-value | Estimate | 95% CI | | p-value |
| **Intercept** | 0.486 | 0.052 | 0.917 | 0.029 | 0.588 | 0.123 | 1.046 | 0.014 | 0.547 | -0.059 | 1.148 | 0.085 |
| **bs(Time_since_diagnosis, 3)1** | -0.013 | -0.632 | 0.618 | 0.967 | -0.225 | -0.953 | 0.524 | 0.548 | 0.379 | -0.735 | 1.524 | 0.515 |
| **bs(Time_since_diagnosis, 3)2** | 0.101 | -0.546 | 0.742 | 0.760 | 0.031 | -0.748 | 0.804 | 0.938 | 0.375 | -0.649 | 1.406 | 0.483 |
| **bs(Time_since_diagnosis, 3)3** | 0.053 | -0.701 | 0.800 | 0.891 | 0.246 | -0.681 | 1.159 | 0.603 | 0.069 | -1.078 | 1.206 | 0.908 |
| **JDM** | 0.006 | -0.412 | 0.426 | 0.979 | -0.118 | -0.642 | 0.416 | 0.665 | 0.250 | -0.458 | 0.959 | 0.503 |
| **Pubertal_Stage = Adolescent** | -0.036 | -0.523 | 0.452 | 0.886 | 0.134 | -0.439 | 0.706 | 0.649 | -0.151 | -1.137 | 0.834 | 0.769 |
| **Pubertal_Stage = Peri-pubertal** | -0.267 | -0.587 | 0.053 | 0.107 | -0.034 | -0.424 | 0.354 | 0.864 | -0.708 | -1.275 | -0.132 | 0.018 |
| **Female** | 0.188 | -0.145 | 0.522 | 0.274 |  |  |  |  |  |  |  |  |
| **Ethnicity = Asian** | -0.411 | -0.893 | 0.072 | 0.099 | -0.298 | -0.902 | 0.305 | 0.338 | -0.743 | -1.525 | 0.034 | 0.074 |
| **Ethnicity = Black** | 0.359 | -0.404 | 1.118 | 0.362 | 0.899 | -0.109 | 1.902 | 0.085 | -0.281 | -1.421 | 0.838 | 0.636 |
| **Ethnicity = Other** | 0.161 | -0.431 | 0.753 | 0.597 | 0.649 | -0.115 | 1.411 | 0.100 | -0.639 | -1.526 | 0.255 | 0.177 |
| **bs(Time_since_diagnosis, 3)1 * JDM** | -0.082 | -0.869 | 0.687 | 0.835 | 0.292 | -0.649 | 1.199 | 0.529 | -0.474 | -1.892 | 0.941 | 0.518 |
| **bs(Time_since_diagnosis, 3)2 * JDM** | -0.467 | -1.451 | 0.526 | 0.356 | -0.531 | -1.717 | 0.666 | 0.386 | -0.630 | -2.301 | 1.024 | 0.464 |
| **bs(Time_since_diagnosis, 3)3 * JDM** | -0.158 | -1.373 | 1.055 | 0.800 | -0.755 | -2.253 | 0.744 | 0.328 | 0.567 | -1.374 | 2.521 | 0.573 |

Table 29: Linear mixed model results for weight Z-scores in the JDM cohort, stratified by pubertal stage at diagnosis of rheumatic disease.

|  | **Pre-Pubertal** | | | | **Peri-Pubertal** | | | | **Adolescents** | | | |
| --- | --- | --- | --- | --- | --- | --- | --- | --- | --- | --- | --- | --- |
|  | Estimate | 95% CI | | p-value | Estimate | 95% CI | | p-value | Estimate | 95% CI | | p-value |
| **Intercept** | 0.305 | -0.235 | 0.845 | 0.271 | -0.121 | -0.866 | 0.610 | 0.750 | 1.466 | -0.204 | 3.133 | 0.097 |
| **bs(Time_since_diagnosis, 3)1** | 0.840 | -0.073 | 1.770 | 0.073 | 0.560 | -0.306 | 1.450 | 0.210 | -0.997 | -2.195 | 0.201 | 0.112 |
| **bs(Time_since_diagnosis, 3)2** | 0.136 | -0.629 | 0.910 | 0.732 | -1.232 | -1.984 | -0.481 | 0.001 | -0.280 | -1.491 | 0.915 | 0.660 |
| **bs(Time_since_diagnosis, 3)3** | 0.242 | -0.639 | 1.121 | 0.593 | 1.365 | 0.524 | 2.194 | 0.002 | -0.115 | -1.408 | 1.174 | 0.865 |
| **JDM** | 0.544 | -0.073 | 1.171 | 0.092 | 0.050 | -0.605 | 0.718 | 0.883 | -1.355 | -2.417 | -0.266 | 0.024 |
| **Female** | -0.161 | -0.557 | 0.239 | 0.430 | 0.660 | 0.063 | 1.254 | 0.032 | -0.223 | -1.708 | 1.244 | 0.769 |
| **Ethnicity = Asian** | -0.570 | -1.149 | 0.007 | 0.059 | -0.550 | -1.535 | 0.432 | 0.281 | -0.128 | -1.529 | 1.265 | 0.865 |
| **Ethnicity = Other** | 0.291 | -0.539 | 1.119 | 0.499 | 0.247 | -0.635 | 1.138 | 0.589 | -0.644 | -2.734 | 1.393 | 0.544 |
| **bs(Time_since_diagnosis, 3)1 * JDM** | -0.672 | -1.856 | 0.470 | 0.253 | -0.574 | -1.610 | 0.435 | 0.268 | 1.996 | 0.324 | 3.623 | 0.020 |
| **bs(Time_since_diagnosis, 3)2 * JDM** | -0.894 | -2.094 | 0.308 | 0.149 | 0.028 | -0.991 | 1.053 | 0.958 | -1.065 | -3.145 | 1.052 | 0.332 |
| **bs(Time_since_diagnosis, 3)3 * JDM** | -0.811 | -2.218 | 0.580 | 0.261 | -0.806 | -1.955 | 0.357 | 0.178 | 4.725 | 1.715 | 7.535 | 0.002 |

Table 30: Baseline characteristics of the Height cohort in patients with observations after the age of 18.

| **Variable** | | **Juvenile Idiopathic Arthritis** | | | **Juvenile Systemic Lupus Erythematosus** | | | **Juvenile Dermatomyositis** | | |
| --- | --- | --- | --- | --- | --- | --- | --- | --- | --- | --- |
|  |  | Control | Cases | p-value | Control | Cases | p-value | Control | Cases | p-value |
| **N (%)** | | 3284 (70.0) | 1409 (30.0) |  | 153 (71.5) | 61 (28.5) |  | 61 (75.3) | 20 (24.7) |  |
| **Age at diagnosis (years)**  **Mean (sd)** | | 12.6 (4.5) | 13.5 (4.1) | <0.001 | 13.7 (4.3) | 14.2 (3.8) | 0.428 | 9.1 (4.7) | 10.8 (5.8) | 0.175 |
| **Pubertal Stage N(%)** | Adolescent | 595 (18.1) | 181 (12.8) | <0.001 | 14 (9.2) | 6 (9.8) | 0.838 | 27 (44.3) | 6 (30.0) | 0.134 |
|  | Peri-pubertal | 1522 (46.3) | 785 (55.7) |  | 90 (58.8) | 38 (62.3) |  | 9 (14.8) | 7 (35.0) |  |
|  | Pre-pubertal | 1167 (35.5) | 443 (31.4) |  | 49 (32.0) | 17 (27.9) |  | 25 (41.0) | 7 (35.0) |  |
| **Ethnicity** | White | 2870 (87.4) | 1228 (87.2) | 0.582 | 128 (83.7) | 46 (75.4) | 0.463 | 52 (85.2) | 17 (85.0) | 0.761 |
|  | Asian | 148 (4.5) | 58 (4.1) |  | 12 (7.8) | 6 (9.8) |  | 5 (8.2) | 1 (5.0) |  |
|  | Black | 60 (1.8) | 22 (1.6) |  | 3 (2.0) | 3 (4.9) |  | 1 (1.6) | 0 (0.0) |  |
|  | Other | 206 (6.3) | 101 (7.2) |  | 10 (6.5) | 6 (9.8) |  | 3 (4.9) | 2 (10.0) |  |
| **Sex** | Male | 592 (18.0) | 261 (18.5) | 0.716 | 18 (11.8) | 12 (19.7) | 0.198 | 10 (16.4) | 4 (20.0) | 0.977 |
|  | Female | 2692 (82.0) | 1148 (81.5) |  | 135 (88.2) | 49 (80.3) |  | 51 (83.6) | 16 (80.0) |  |
| **Time since diagnosis at first height measurement (years)**  **Median (IQR)** | | 6.2 (4.5) | 5.2 (4.2) | <0.001 | 5.0 (4.4) | 4.4 (3.9) | 0.319 | 9.7 (4.8) | 7.8 (5.6) | 0.146 |
| **Final Height (cm)**  **Mean (sd)** | | 167.1 (8.6) | 166.7 (8.8) | 0.145 | 166.4 (8.0) | 165.7 (8.7) | 0.555 | 167.2 (7.7) | 166.6 (8.5) | 0.759 |
| **Final Height Z score**  **Mean (sd)** | | 0.2 (1.1) | 0.1 (1.2) | 0.041 | 0.2 (1.1) | -0.1 (1.1) | 0.075 | 0.2 (1.1) | 0.0 (1.2) | 0.519 |

Table 31: Baseline characteristics of the Weight cohort in patients with observations after the age of 18

| **Variable** | | **Juvenile Idiopathic Arthritis** | | | **Juvenile Systemic Lupus Erythematosus** | | | **Juvenile Dermatomyositis** | | |
| --- | --- | --- | --- | --- | --- | --- | --- | --- | --- | --- |
|  |  | Control | Cases | p-value | Control | Cases | p-value | Control | Cases | p-value |
| **N (%)** | | 4930 (67.3) | 2392 (32.7) |  | 246 (66.0) | 127 (34.0) |  | 91 (67.9) | 43 (32.1) |  |
| **Age at diagnosis (years)**  **Mean (sd)** | | 12.9 (4.4) | 13.8 (4.0) | <0.001 | 14.2 (4.0) | 14.8 (3.3) | 0.152 | 9.4 (4.7) | 10.7 (5.1) | 0.143 |
| **Pubertal Stage N (%)** | Adolescent | 782 (15.9) | 251 (10.5) | <0.001 | 18 (7.3) | 6 (4.7) | 0.468 | 35 (38.5) | 12 (27.9) | 0.228 |
|  | Peri-pubertal | 2489 (50.5) | 1417 (59.2) |  | 153 (62.2) | 86 (67.7) |  | 18 (19.8) | 14 (32.6) |  |
|  | Pre-pubertal | 1659 (33.7) | 724 (30.3) |  | 75 (30.5) | 35 (27.6) |  | 38 (41.8) | 17 (39.5) |  |
| **Ethnicity** | White | 4343 (88.1) | 2099 (87.8) | 0.905 | 209 (85.0) | 91 (71.7) | 0.003 | 77 (84.6) | 37 (86.0) | 0.476 |
|  | Asian | 204 (4.1) | 97 (4.1) |  | 15 (6.1) | 16 (12.6) |  | 10 (11.0) | 2 (4.7) |  |
|  | Black | 75 (1.5) | 41 (1.7) |  | 2 (0.8) | 7 (5.5) |  | 1 (1.1) | 1 (2.3) |  |
|  | Other | 308 (6.2) | 155 (6.5) |  | 20 (8.1) | 13 (10.2) |  | 3 (3.3) | 3 (7.0) |  |
| **Sex** | M | 678 (13.8) | 368 (15.4) | 0.066 | 20 (8.1) | 16 (12.6) | 0.23 | 15 (16.5) | 13 (30.2) | 0.11 |
|  | F | 4252 (86.2) | 2024 (84.6) |  | 226 (91.9) | 111 (87.4) |  | 76 (83.5) | 30 (69.8) |  |
| **Time since diagnosis at first weight measurement (years)**  **Median (IQR)** | | 5.7 (4.4) | 4.8 (4.1) | <0.001 | 4.5 (4.0) | 3.8 (3.3) | 0.092 | 9.3 (4.8) | 8.0 (5.2) | 0.164 |
| **Weight (kg)**  **Mean (sd)** | | 65.4 (14.8) | 66.6 (15.6) | 0.001 | 65.8 (16.5) | 65.7 (16.2) | 0.931 | 63.7 (13.0) | 64.6 (17.0) | 0.751 |
| **Weight Z-score** | | 0.5 (1.4) | 0.5 (1.4) | 0.014 | 0.5 (1.5) | 0.5 (1.5) | 0.744 | 0.3 (1.3) | 0.1 (1.6) | 0.476 |
